# Genome-wide identification of three-amino-acid-loop-extension gene family and their expression profile under hormone and abiotic stress treatments during stem development of *Prunus mume*

**DOI:** 10.3389/fpls.2022.1006360

**Published:** 2022-09-23

**Authors:** Qingqing Yang, Cunquan Yuan, Tianci Cong, Jia Wang, Qixiang Zhang

**Affiliations:** Beijing Key Laboratory of Ornamental Plants Germplasm Innovation and Molecular Breeding, National Engineering Research Center for Floriculture, Beijing Laboratory of Urban and Rural Ecological Environment, Engineering Research Center of Landscape Environment of Ministry of Education, Key Laboratory of Genetics and Breeding in Forest Trees and Ornamental Plants of Ministry of Education, School of Landscape Architecture, Beijing Forestry University, Beijing, China

**Keywords:** *Prunus mume*, TALE transcription factors, stem development, abiotic stress, hormone response

## Abstract

Transcription factors encoded by the three-amino-acid-loop-extension (TALE) gene family play a key role in regulating plant growth and development, and are involved in plant hormone regulatory pathways and responses to various environmental stresses. Researchers are currently studying *TALE* genes in different species, but *Prunus mumeTALE* genes have not yet been studied. Therefore, based on the *P. mume* genome, we found a total of 23 *TALE* gene family members, which were distributed on eight chromosomes. *TALE* genes contained the characteristic domains of this family, and could be divided into KNOTTED-like homeobox (KNOX) subfamily and BEL1-like homeobox (BELL) subfamily. They can form heterodimers with each other. Fragment duplication and tandem duplication events were the main reasons for the expansion of *P. mumeTALE* gene family members and the *TALE* genes were selected by different degrees of purification. The inter-species collinearity analysis showed that the relationship between *P. mume* and other four *Prunus* species was consistent with the distance of origin. Eleven members of *P. mumeTALE* genes were specifically highly expressed in stem, mainly at the early stage of stem development. The cis-element analysis showed that the promoter of *P. mumeTALE* genes contained a variety of hormone and abiotic stress response elements, and four *TALE* genes responded to two kinds of abiotic stresses and four kinds of hormones at the early stage of stem development. In conclusion, this study lays a foundation to explore the role of *TALE* gene family in *P. mume* growth and development.

## Introduction

Transcription factors (TFs) encoded by homeobox genes contain many members and play a key role in regulating plant growth and development (Chan et al., 1998). Vollbrecht et al. (1991) discovered a homeobox gene, maize *Knotted-1 (KN1)*, for the first time in the plant kingdom. The *kn1* mutations caused by insertion or tandem duplication of transposable elements significantly affected leaf development (Vollbrecht et al., 1991). The homeobox domain present in homeobox genes consists of 60 amino acids, forming three helical structures. The first helix and the second helix form a ring, while the second helix and the third helix form helix-turn-helix (Billeter et al., 1993). The predecessors classified Homeobox genes according to the sequence features of the homeobox domain. Originally, Homeobox genes were divided into seven categories, including *Arabidopsis thaliana* homeobox 8 (ATHB8), BEL1-like homeobox (BELL/BLH), GL2, homeobox from *A. thaliana* 1 (HAT1), homeobox from *A. thaliana* 2 (HAT2), KNOTTED-like homeobox (KNOX/KNAT), and *Zea mays* homeobox (ZM-HOX; Bharathan et al., 1997). Later, Mukherjee et al. classified them into 14 categories, including BELL/BLH, DDT, homeodomain-leucine zipper I to IV (HD-ZIP I to IV), KNOX/KNAT, luminidependens (LD), nodulin homeobox genes (NDX), plant homeodomain (PHD), PINTOX, plant zinc finger (PLINC), SAWADEE, and wuschel homeobox (WOX; Mukherjee et al., 2009). In 2017, Jin et al. reduced the homeobox genes into five categories in plant TF database PlantTFDB 4.0, including HD-ZIP, TALE, WOX, HB-PHD, and HB-others (Jin et al., 2017). Among them, TALE includes the two types of gene subfamilies BELL and KNOX, mentioned above, because the homeobox domain of these two types of genes contains three additional amino acids, which can form an additional loop connection to construct three-amino-acid-loop-extension (TALE) homeobox domain (Hamant and Pautot, 2010).

*KNOTTED*-like homeobox-like genes play key regulatory roles in plant meristem and leaf development. Such gene-encoded proteins typically contain four domains, including homeobox, KNOX1, KNOX2, and ELK. At first, *KNOX* genes were divided into two categories according to their expression and function, namely class I and II. Compared with class I, the gene expression range of class II is wider (Hake et al., 2004; Gao et al., 2015). Interestingly, later studies found that *KNOX A. thaliana MEINOX protein (KNATM)* only contained the KNOX domain, which was classified as a new group (class III) by phylogenetic analysis. *KNATM* is only found in eudicots, and some species have multiple paralogs (Magnani and Hake, 2008). *Arabidopsis thaliana* KNOX class I consists of four members: *shoot meristemless (STM)*, *KNAT1/BP*, *KNAT2*, and *KNAT6*, which are mainly expressed in meristem and stem, and their ectopic expression in leaves can significantly affect leaf phenotype. *STM* is a meristem marker gene and also the first *KNOX* gene expressed during embryonic development (Scofield and Murray, 2006; Shani et al., 2009; Hay and Tsiantis, 2010). It affects the development and maintenance of SAM, and the formation of AM during postembryonic development (Shi et al., 2016). *KNAT1/BP* and *KNAT6* have redundant functions with *STM* in maintaining SAM activity and organ separation. There is partial duplication of chromosome segments between *KNAT2* and *KNAT6*, and single gene mutation does not significantly affect shoot development (Bharathan et al., 2002; Belles-Boix et al., 2006). *KNAT1/BP* can restrict the expression of *KNAT6* and *KNAT2* during the reproductive stage, allowing normal inflorescence development (Ragni et al., 2008). *Arabidopsis thaliana* KNOX class II contains four members: *KNAT3*, *KNAT4*, *KNAT5*, and *KNAT7*. Studies have shown that *KNAT7* and its homologous genes affect the formation of lignin, thereby regulating the development of secondary cell wall (SCW). KNAT3 can form heterodimers with KNAT7, thus jointly promoting the formation of SCW (Li et al., 2012; Ma et al., 2019; Wang et al., 2019b). In addition, *KNAT3*, *KNAT4*, and *KNAT5* play regulatory roles in *A. thaliana* root development with functional redundancy (Truernit and Haseloff, 2007). *Medicago truncatula KNAT3/4/5*-like genes regulate the development of symbiotic nodules (Di Giacomo et al., 2017). *KNATM* is expressed in the proximal domain of organ primordia and at the border of mature organs. Although it does not have a homeobox domain, it can still function as a transcriptional regulator. In addition, KNATM can interact with KNAT1/BP through the acidic coiled-coil domain (Magnani and Hake, 2008).

*BEL1*-like homeobox-like genes encoded proteins contain two conserved domains, namely homeobox and POX. *Arabidopsis thaliana* *BELL* genes has 12 members, including *BEL1*, *A. thaliana homeobox 1 (ATH1)*, *BLH1-10*, and there is no clear classification system. In addition, BELL proteins frequently interact with KNOX proteins to perform their functions (Byrne et al., 2003). For example, KNAT1/BP can form heterodimers with PENNYWISE (PNY)/BLH9 to regulate early developmental events in inflorescence meristem (Smith and Hake, 2003). ATH1 can interact with STM to activate the expression of *STM*, so that *STM* forms a self-activating loop, thereby maintaining the activity of SAM (Cao et al., 2020). SAWTOOTH1 (BLH2/SAW1) and SAWTOOTH2 (BLH4/SAW2) can interact with KNAT1/BP, KNAT2, KNAT5, and STM, and regulate leaf edge development to a certain extent by inhibiting the expression of one or more *KNOX* genes (Kumar et al., 2007). BLH6 can interact with KNAT7, and this interaction enhances the inhibition ability of *BLH6* and *KNAT7*. In addition, *BLH6* and *KNAT7* can directly inhibit the expression of *REVOLUTA (REV)* to regulate the formation of SCW (Liu et al., 2014). Recently, researchers have studied the interaction between TALE proteins in tomato and identified 75 pairs of KNOX-BLH interactions. It was found that the interaction between KNOX class I member SlKN5 and BELL proteins (SlBLH5 and SlBLH7) played an important regulatory role in the process of fruit differentiation (Ezura et al., 2022).

Previous studies have found that the TALE gene family is involved in plant hormone regulatory pathways (Tsuda and Hake, 2015; Niu and Fu, 2022). The exogenous hormone application showed that the expression of KNOX gene family members of *Caucasian clover* could respond to the changes of 6-BA, IAA, and KT signals (Zhang et al., 2022b). Ectopic expression of the maize *KNOX*-like gene *KN1* in leaves enhances auxin signaling (Bolduc et al., 2012), and *KN1* also can activate *ga2ox1* expression by binding to its intron, which in turn negatively regulates gibberellin (GA) accumulation, thereby maintaining meristematic cell identity (Bolduc and Hake, 2009). Overexpression of tomato *KNOX*-like gene *Tkn4* increases seedling sensitivity to GA and auxin, and increases the expression of genes related to GA and auxin synthesis (Yan et al., 2019). Ectopic expression of auxin synthesis gene in *A. thaliana* leaf axils can downregulate the expression of *STM* (Wang et al., 2015) and *A. thaliana* *KNOX* gene can rapidly activate CK biosynthetic gene expression, which is important for maintaining normal SAM development (Jasinski et al., 2005; Yanai et al., 2005). Infection of *A. thaliana* leaves by *Rhodococcus fascians* can cause local CK responses and reduce GA signaling, which may provide a suitable environment for the expression of *KNOX*-like genes, resulting in the formation of leaf edge serrations (Depuydt et al., 2008). *Arabidopsis thaliana BLH1* can form a heterodimer with *KNAT3* to activate the expression of abscisic acid (ABA) response gene *ABSCISIC ACID-INSENSITIVE 3 (ABI3)*, which promotes plant response to ABA during seed germination and seedling stages (Kim et al., 2013). *MdKNOX19*, a member of the apple KNOX subfamily, is an ABA-responsive gene. Overexpression of *MdKNOX19* increases the ABA sensitivity of apple callus, and can directly activate the expression of *MdABI5*, a key gene in the ABA signaling pathway (Jia et al., 2021). The rice *KNOX*-like gene *homeobox1 (OSH1)* can activate brassinosteroid (BR) degradation-related genes to inhibit the BR pathway, and the loss of *OSH1* function leads to the increase of BR level (Tsuda et al., 2014). Litchi *LcKNAT1* can inhibit the expression of ethylene biosynthesis genes by directly binding to their promoters, thereby inhibiting fruit abscission (Zhao et al., 2020).

Furthermore, TALE gene family members are not only involved in hormonal regulatory pathways but also in response to a variety of abiotic stresses (Hao et al., 2021). The promoters of TALE gene family members in soybean contained cis-elements that responded to various stresses, and the expression level of *GmTALE* genes could change in response to salt stress and drought stress (Wang et al., 2021). Overexpression of the *KNOX*-like gene *TaKNOX11-A* of *Triticum aestivum* in *A. thaliana* can increase the content of proline and reduce the content of malondialdehyde, thereby enhancing the salt tolerance and drought resistance of the plant (Han et al., 2022). Cotton *BELL*-like gene *GhBLH5-A05* plays an important role in combating drought stress. Silencing *GhBLH5-A05* by virus induced gene silencing (VIGS) technology reduced the drought resistance of cotton, and overexpression of *GhBLH5-A05* could increase the expression level of drought-responsive genes, thereby increasing the drought resistance of cotton (Zhang, 2021). In addition, *GhBLH5-A05* can enhance its own function through protein interaction with *KNOX*-like gene *GhKNAT6-A03* (Zhang et al., 2021b). *Glycine max H1 Sbh1 (GmSBH1)*, a member of the soybean KNOX subfamily, has a key regulatory role in response to high temperature and humidity (HTH) stress. At the same time, GmSBH1 protein can form a heterodimer with GmBLH4 to participate in the response to HTH stress during seed development (Shu et al., 2015; Tao et al., 2018). *Populus alba* × *P. glandulosa* KNOX subfamily member *PagKNAT2/6b* can be significantly induced by drought treatment, and can mediate drought response by down-regulating *PagGA20ox1* gene of GA pathway (Song et al., 2021). Members of the pear KNOX subfamily can respond to drought stress treatment, specifically, the expression of *PbKNOX7/13* is increased under drought stress, while the expression of *PbKNOX5/16* is inhibited under drought stress (Liu et al., 2022).

*Prunus mume* belongs to genus *Prunus* of *Rosaceae* and its TALE gene family has not yet been studied, but the research on the *KNOX* genes of other *Prunus* plants has been reported. Misexpression of peach *KNOPE1* (belonging to KNOX class I) may be involved in leaf hyperplasia caused by leaf curl disease (Testone et al., 2008). In addition, *KNOPE1* is expressed in cortex and procambium at the primary growth stage of stems and prevents the lignification of primary stems by inhibiting lignin-related genes (Testone et al., 2012). Subsequent studies found that *KNOPE1* could inhibit the expression of *PpGA3ox1* to affect gibberellin(GA) homeostasis, thereby regulating peel differentiation (Testone et al., 2015). Peach *KNOPE3* (belonging to KNOX class II) may be involved in the regulation of sugar transport processes (Testone et al., 2009). The members of the KNOX subfamily of pear have an important regulatory effect on the growth and development of pear. Specifically, they are highly expressed in robust hybrid progeny and weakly expressed in dwarf hybrid progeny (Liu et al., 2022). In addition, *PbKNOX* in pear can inhibit the level of lignin, thereby regulating the formation of stone cells in fruit (Cheng et al., 2019). The transformation of maize *KNOX1* into plum can significantly improve the regeneration efficiency of plum explants (Srinivasan et al., 2011).

In this study, we used *P. mume* as experimental material to identify members of the TALE gene family based on genome-wide data, and performed gene and protein structure analysis, phylogenetic analysis, chromosome location analysis, collinearity analysis, promoter element prediction, and protein interaction prediction. At the same time, the response of *TALE* genes to hormones and abiotic stresses was explored during the early development of stem, thus laying a foundation for the functional study of the *P. mume* TALE gene family.

## Materials and methods

### Identification and gene structure analysis of TALE gene family members in *Prunus mume*

We obtained the genome information of *P. mume* (Zhang et al., 2012) on NCBI,^1^ and used Pfam (http://pfam.xfam.org; Mistry et al., 2021) to download the seed sequences of POX, KNOX1, KNOX2, and ELK domains. The genes containing at least one of the above domains (required *E*-value>10^–5^) were obtained from the *P. mume* genome with the help of hmmer 3.0 (Johnson et al., 2010) and defined as members of the *P. mume* TALE gene family. We used the GSDS website (http://gsds.gao-lab.org/index.php; Hu et al., 2015) to draw the intron-exon structure map of the TALE gene family members.

### Basic information of TALE protein sequences in *Prunus mume*

The isoelectric point (pI), molecular weight (MW), overall average hydropathic coefficient (GRAVY), and instability coefficient of the TALE gene family members were analyzed using the EXPASY website (https://web.expasy.org/protparam; Wilkins et al., 1999). Transmembrane domain analysis was performed using the TMHMM website.^2^ Subcellular localization prediction was performed using the WoLF PSORT website (https://wolfpsort.hgc.jp; Horton et al., 2007). Protein secondary structure prediction was performed using the SOPMA website (https://npsa-prabi.ibcp.fr/cgi-bin/npsa_automat.pl?page=npsa_sopma.html; Geourjon and Deleage, 1995). Protein phosphorylation sites were predicted using the NetPhos website (https://services.healthtech.dtu.dk/service.php?NetPhos-3.1; Blom et al., 2004).

### Phylogenetic analysis and conserved motif analysis of TALE protein sequences in *Prunus mume*

In order to elucidate the phylogenetic relationship of *TALE* genes in *P. mume*, the TALE gene family in the genome of two other *Prunus* species *(P. armeniaca, P. persista)* and the model plant *A. thaliana* was analyzed together. The sequences of the *A. thaliana* TALE gene family were derived from UniProt (https://www.uniprot.org/blast; Bateman et al., 2021). The genomes of *P. armeniaca* and *P. persista* were downloaded from NCBI (see footnote 1). MEGA7.0 [neighbor-joining method (NJ), 1,000 Bootstrap repeats] were used to construct the TALE gene family evolution tree of *P. mume, P. armeniaca, P. persista*, and *A. thaliana*, so as to predict the homologous genes of the *A. thaliana* TALE gene family in three kinds of *Prunus* species.

Motif prediction was performed on the amino acid sequences of the TALE gene family of *P. mume* using the MEME website (https://meme-suite.org/meme/tools/meme; Bailey et al., 2015), using the default parameters, except that the number of motifs was set as 6, and the motif structure map was mapped using TBtools (Chen et al., 2020). Pfam’s batch sequence search tool (http://pfam.xfam.org/search#tabview=tab1; Mistry et al., 2021) was used to align the amino acid sequences of the TALE gene family of *P. mume* with known domains, and TBtools were used to map and beautify the analysis results (Chen et al., 2020), followed by analysis of the corresponding relationship between the motif prediction results and the structural domain alignment results.

### Chromosomal location and collinearity analysis of TALE gene family members in *Prunus mume*

The MG2C website^3^ was used to analyze the chromosomal location of the *P. mume* TALE gene family. The intra-species collinearity analysis of the *P. mume* TALE gene family was performed using MCScanX (Wang et al., 2012, 2013), and the non-synonymous substitution (Ka) and synonymous substitution (Ks) values of the collinear pair were calculated with the help of the PAL2NAL website (http://www.bork.embl.de/pal2nal; Suyama et al., 2006). The evolution time of collinear pairs was predicted using the formula T = Ks/2λ × 10^6^ Mya (λ = 1.5 × 10^−8^ for dicots; Wang et al., 2019a). We selected four other *Prunus* species, including *P. armeniaca*, *P. persica*, *P. avium*, and *P. dulcis*, and used MCScanX (Wang et al., 2012, 2013) to analyze the inter-species collinearity between *P. mume* and the above four species. The genomes of the other four plants (Ezhova, 2003; Alioto et al., 2020; Pinosio et al., 2020; Zhang et al., 2021a) were downloaded from NCBI (see footnote 1) and the Ensembl Plants website.^4^

### Promoter analysis and protein interaction prediction of TALE gene family members in *Prunus mume*

The 2000 bp promoter sequence upstream of ATG of the TALE gene family of *P. mume* was extracted by TBtools (Chen et al., 2020), and the cis-acting elements on the promoter sequence were analyzed by the PlantCARE website (http://bioinformatics.psb.ugent.be/webtools/plantcare/html; Rombauts et al., 1999). The STRING website (https://cn.string-db.org; Szklarczyk et al., 2021) was used to predict the protein interactions between members of the TALE gene family, and Cytoscape was used to map protein interaction networks (Shannon et al., 2003).

### Analysis of expression patterns of the TALE gene family members in *Prunus mume*

Tissue-specific expression analysis of TALE gene family members in *P. mume*: The expression profiles of *TALE* genes in five different tissues (root, stem, leaf, flower bud, and fruit) were mapped using previous transcriptome data (GEO No. GSE40162), and *P. mume* wild species in Tongmai town, Tibet, China (accession No. BJFU1210120008) was used in this previous study (Zhang et al., 2012). TBtools was used to draw the heatmap with parameters set to row scale and scale size by area (Chen et al., 2020).

Expression profile analysis of *P. mume* *TALE* genes at different stem developmental stages: The plant material *P. mume* cv. “Jiangmei” used in this study was planted on the campus of Beijing Forestry University. To explore the effect of the TALE gene family on stem development, annual stems (that is, 1-year-old branches) with the same growth length (about 20 cm) were obtained from the same tree in May. The three developmental stages of the stem were divided as follows: the first developmental stage included the apical bud and the first to third stem nodes counting down from the apical bud of each annual stem (leaves removed), the second developmental stage included the fourth to sixth stem nodes of each annual stem (leaves removed), and the third developmental stage included the seventh to ninth stem nodes of each annual stem (leaves removed). Samples for each developmental stage were obtained in three replicates. All samples were snap-frozen in liquid nitrogen and stored in a −80°C freezer.

Expression profile analysis of *P. mume* *TALE* genes under hormone or abiotic stress treatments: In order to explore the response of the TALE gene family to exogenous hormones and abiotic stresses in early stem development, annual stems with the same growth length (20 cm) were obtained from the same tree in May, and the annual stems were treated with four hormones and two stresses, then the first developmental stage samples of the treated annual stems were used for the fluorescence quantitative assay. Various treatments were carried out by inserting annual stems into MS liquid medium containing different hormones or stress treatment-related substances. Each liter of MS liquid medium contains 4.43 g MS powder, 25 g sucrose and 7 g agar. Hormone treatments included 1 mmol/L 6-BA, 1 mmol/L NAA, 1 mmol/L GA3, and 1 mmol/L ABA. Abiotic stress included 300 mmol/L NaCl and 300 mmol/L mannitol. The treatment time included 1, 3, 6, and 12 h, and the untreated (that is, 0 h) first developmental stage samples of the annual stems were the blank control. The test was carried out in an incubator, and the setting conditions were 16 h (25°C) during the day, 8 h (18°C) at night, and 80% humidity. Each treatment experiment was repeated three times. All samples were snap-frozen in liquid nitrogen and stored in a −80°C freezer.

### RNA extraction and qRT-PCR

RNA was extracted from samples by RNA Extraction Kit (Takara, Dalian, China), the residual DNA in each sample was removed by DNase I, and the first strand of cDNA was synthesized by PrimeScript RT reagent kit (Takara, Dalian, China). Primer design was completed by NCBI primer tool and the primers were synthesized by Beijing Qingke Biotechnology Co., Ltd. (Supplementary Table S1). qRT-PCR was performed with SYBR premix ex Taq II Kit (Takara, Dalian, China). The total reaction system was 20 μl: SYBR Mix 10 μl, 10-fold diluted cDNA template 1 μl, 0.4 μl of forward and reverse primers (10 μmol/L) respectively, and H2O 8.2 μl. The reaction procedure is as follows: pre denaturation at 95°C for 3 min; denaturation at 95°C for 10 s, renaturation at 60°C for 1 min, 40 cycles; fluorescence collection at 60°C. *Actin* was used as the internal reference gene in the experiment (Xu et al., 2015). Three technical repeats were set up and the gene expression level was calculated by 2^–ΔΔCt^ (Schmittgen and Livak, 2008). Microsoft Office Excel was used for data analysis and histogram plotting.

## Results

### Identification and gene structure analysis of TALE gene family members in *Prunus mume*

In order to obtain members of the TALE gene family in *P. mume*, we used the seed sequences of POX, KNOX1, KNOX2, and ELK domains downloaded from Pfam to align and analyze the *P. mume* genome, and the genes containing at least one of the above domains were defined as members of the TALE gene family. Finally, 23 *P. mume* *TALE* genes were obtained, and according to the order number of the genes in the genome, they were named *PmTALE1–PmTALE23* in order from small to large.

**Figure 1:**
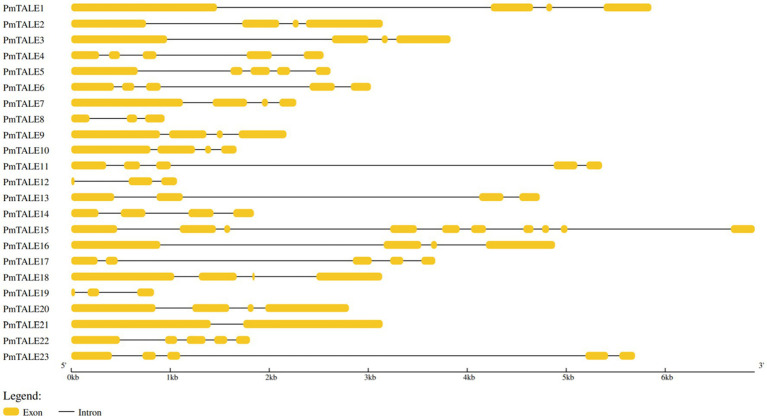
Schematic diagram of the exon-intron structure of the three-amino-acid-loop-extension (TALE) gene family members in *Prunus mume.*

Analysis of the basic information of the *P. mume* TALE gene family showed that a total of 12 members of the family were located on the sense strand, and the other 11 members were located on the antisense strand. The gene length of *PmTALE19* (837 bp) and the length of its coding region (327 bp) were the shortest. The length of *PmTALE15* (6,905 bp) and the coding region of *PmTALE1* (2,445 bp) were the longest. All members of the *P. mume* TALE gene family except *PmTALE15* and *PmTALE21* genes contain 4–5 exons and 3–4 introns, while *PmTALE15* contains 10 exons and nine introns and *PmTALE21* contains only two exons and one intron (Table 1). Gene structure analysis showed that compared with other members, *PmTALE1*, *PmTALE11*, *PmTALE13*, and *PmTALE23* all contained a longer intron with lengths of 2,766, 3,869, 2,995, and 4,094 bp respectively, while *PmTALE18* contained the shortest exon with a length of only 24 bp (Figure 1).

**Table 1:** Basic information of *Prunus mume* TALE gene family members.

Gene name	Gene ID	Start	End	Gene length (bp)	ORF length (bp)	Introns number	Exons number	locus	strand
*PmTALE1*	Pm001057	6,754,856	6,760,716	5,861	2,445	3	4	1	−
*PmTALE2*	Pm001514	10,884,167	10,887,314	3,148	1,968	3	4	1	+
*PmTALE3*	Pm002609	20,525,180	20,529,012	3,833	1,947	3	4	1	+
*PmTALE4*	Pm003159	23,453,653	23,456,202	2,550	987	4	5	1	−
*PmTALE5*	Pm004674	6,438,386	6,441,005	2,620	1,233	4	5	2	−
*PmTALE6*	Pm005456	11,142,568	11,145,595	3,028	1,161	4	5	2	−
*PmTALE7*	Pm005487	11,294,946	11,297,219	2,274	1,707	3	4	2	−
*PmTALE8*	Pm005886	13,532,612	13,533,555	944	489	2	3	2	+
*PmTALE9*	Pm006290	15,915,639	15,917,813	2,175	1,818	3	4	2	+
*PmTALE10*	Pm006291	15,920,256	15,921,926	1,671	1,398	3	4	2	+
*PmTALE11*	Pm006717	18,638,511	18,643,873	5,363	1,062	4	5	2	+
*PmTALE12*	Pm006718	18,642,805	18,643,873	1,069	432	2	3	2	+
*PmTALE13*	Pm009705	521,896	526,629	4,734	1,152	3	4	3	−
*PmTALE14*	Pm015136	17,650,332	17,652,177	1,846	990	3	4	4	−
*PmTALE15*	Pm018677	19,956,308	19,963,212	6,905	1,971	9	10	5	−
*PmTALE16*	Pm021823	11,579,872	11,584,759	4,888	2,040	3	4	6	−
*PmTALE17*	Pm023742	7,826,429	7,830,106	3,678	852	4	5	7	+
*PmTALE18*	Pm023924	9,275,546	9,278,687	3,142	2,115	3	4	7	+
*PmTALE19*	Pm025046	15,732,599	15,733,435	837	327	2	3	7	−
*PmTALE20*	Pm026023	7,211,008	7,213,813	2,806	2,133	3	4	8	+
*PmTALE21*	Pm026549	10,466,095	10,469,241	3,147	1,851	1	2	8	−
*PmTALE22*	Pm027656	16,386,768	16,388,573	1,806	1,077	4	5	8	+
*PmTALE23*	Pm028537	110,304	115,999	5,696	1,065	4	5	scaffold1397	+

### Basic information analysis of TALE protein sequences in *Prunus mume*

**Figure 2:**
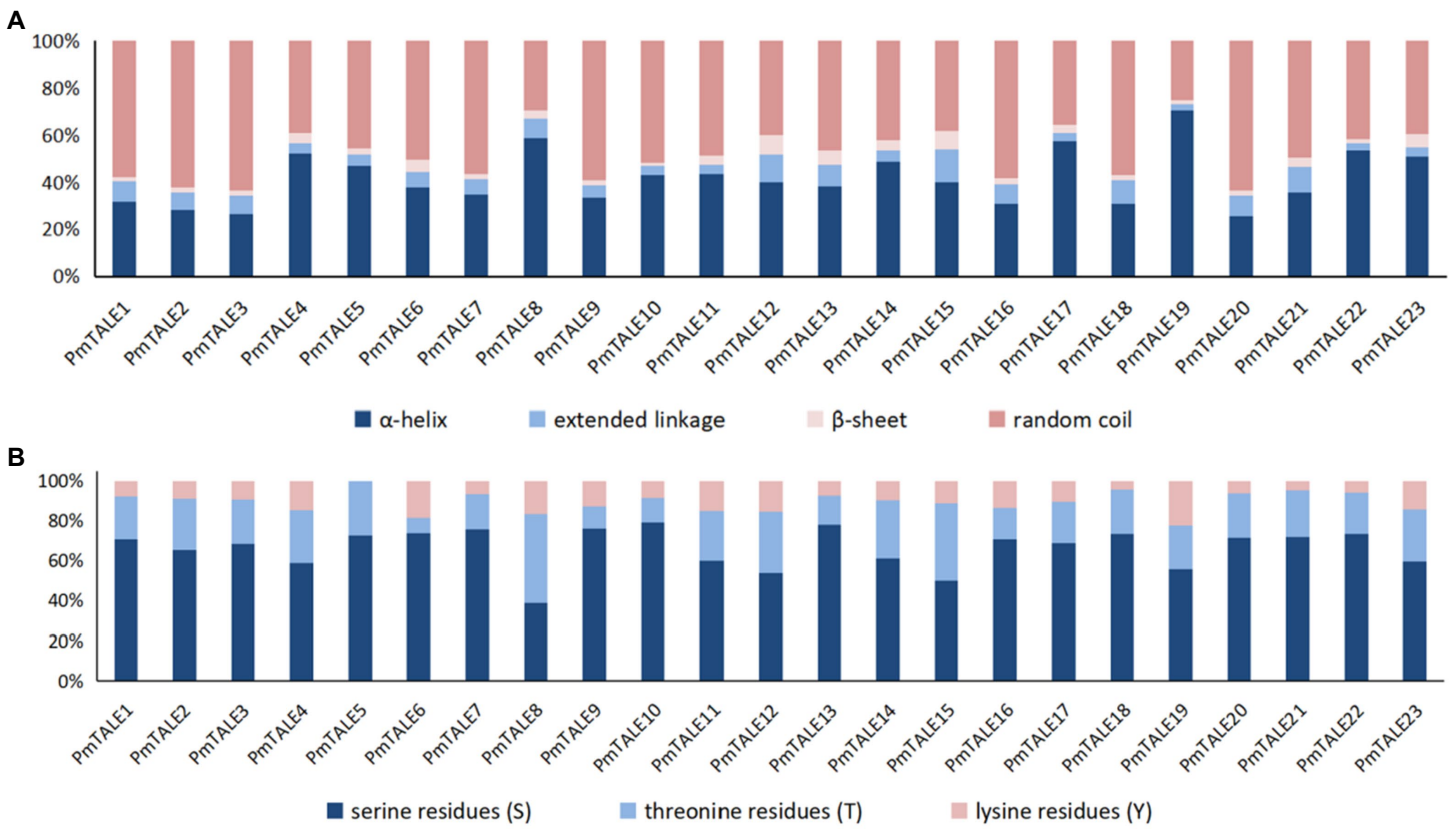
Analysis of protein secondary structure and phosphorylation site of *Prunus mume* TALE gene family members, **(A)** reflects the proportion of the four types of secondary structures of the TALE proteins; **(B)** reflects the proportion of the three types of phosphorylation sites of the TALE proteins. S, serine residues; T, threonine residues; and Y, lysine residues.

Analysis of the basic information of the *P. mume* TALE gene family proteins showed that the number of amino acids ranged from 108 to 814. Among them, PmTALE19 protein sequence has the shortest length and the smallest molecular weight, which is 12042.49 Da. PmTALE1 protein sequence has the longest length and the largest molecular weight, which is 89285.16 Da. The isoelectric points of PmTALE1 and PmTALE2 proteins are 7.17 and 7.21 respectively, showing near neutral. The isoelectric point of PmTALE15 protein is 9.52, showing its alkaline nature. The protein isoelectric points of other 20 gene family members are all less than 7, showing their acidic nature. The GRAVY of all gene family proteins was negative, and the instability coefficients were all greater than 40, indicating that all proteins were soluble and the protein structures were unstable. The prediction results of the transmembrane domain showed that there was no transmembrane helical region in all protein amino acid sequences, so they were all distributed outside the membrane. Subcellular localization prediction results showed that all proteins were localized in the nucleus, suggesting their function as transcription factors (Supplementary Table S2). The secondary structures of all proteins were similar, with a high proportion of α-helix and random coil, a small proportion of β-sheet and extended linkage (Figure 2A). In addition, the prediction results of protein phosphorylation sites show that there are various phosphorylation sites on the amino acid sequence of all members, among which serine residues (S) account for the highest proportion, while threonine residues (T) and lysine residues (Y) account for a small proportion, indicating that the biological function of *P. mume* TALE gene family members may be regulated by kinases (Figure 2B).

### Phylogenetic analysis and conserved motif analysis of TALE protein sequence in *Prunus mume*

**Figure 3:**
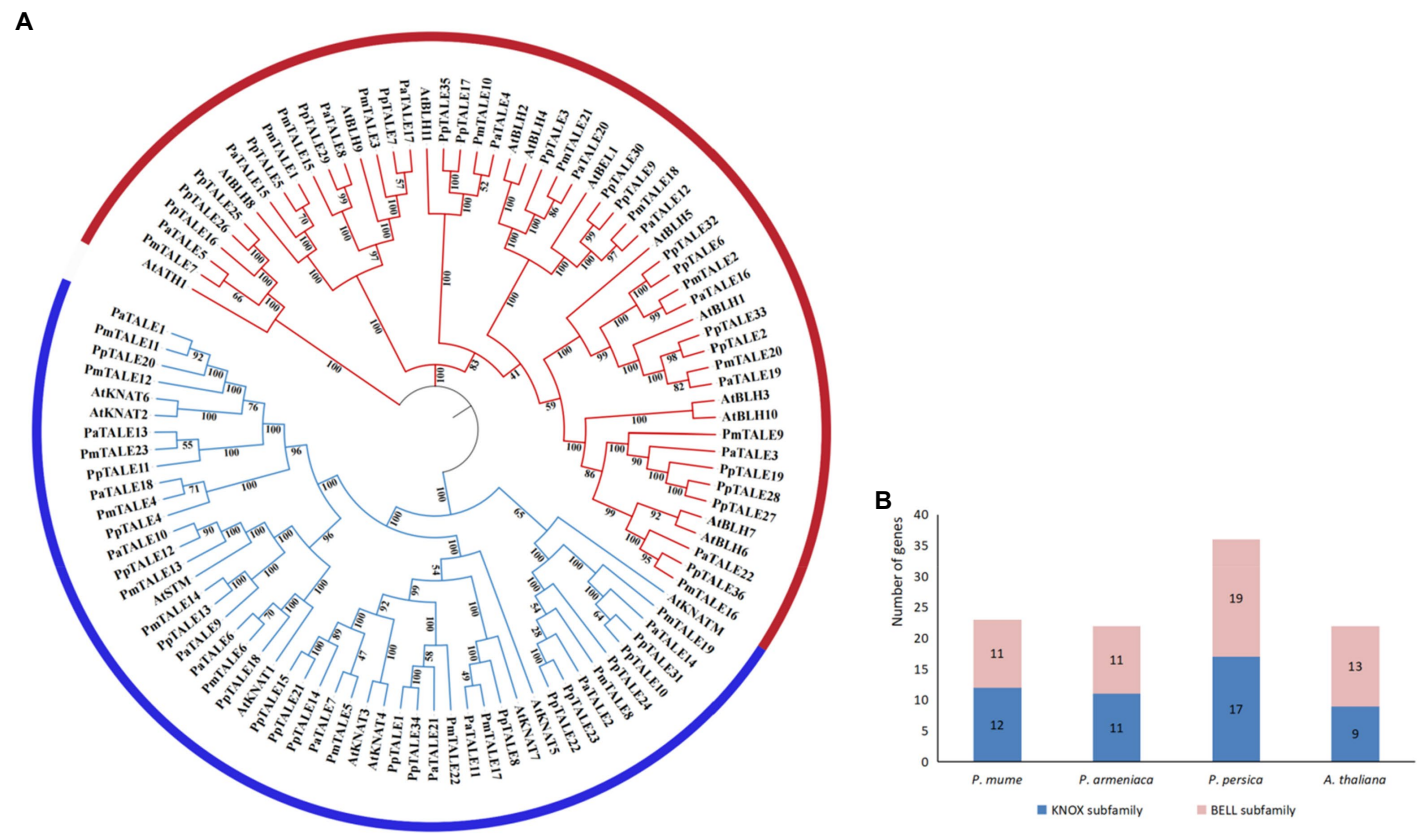
Phylogenetic analysis of TALE gene family protein sequences in *Prunus mume* and other plant species, **(A)** shows phylogenetic tree of TALE gene family protein sequences in *P. mume, Prunus armeniaca, Prunus persica*, and *Arabidopsis thaliana.* PmTALE represents the *P. mume* TALE protein, PaTALE represents the *P. armeniaca* TALE protein, PpTALE represents the *P. persica* TALE protein, and the rest represent the *A. thaliana* TALE proteins. On the periphery of the figure, the blue semi-circle represents the KNOTTED-like homeobox (KNOX) subfamily, and the red semi-circle represents the BEL1-like homeobox (BELL) subfamily; **(B)** shows the gene numbers of subfamily in *P. mume, P. armeniaca, P. persica*, and *A. thaliana.*

In order to predict the phylogenetic relationship of TALE gene family members in *P. mume*, the phylogenetic tree was constructed from the protein sequences of TALE gene family members in *P. mume*, two other *Prunus* species (*P. armeniaca and P. persica*) and *A. thaliana*. The results showed that the TALE gene families of these four species can all be divided into two subfamilies (Figure 3A), and the number of members of the two subfamilies is not much different (Figure 3B), indicating that the overall evolutionary relationships within the TALE gene families of these three *Prunus* species are all similar to *A. thaliana*. According to the clustering results of *P. mume* and *A. thaliana*, PmTALE1-3, PmTALE7, PmTALE9-10, PmTALE15-16, PmTALE18, and PmTALE20-21, a total of 11 members clustered with the *A. thaliana* BELL subfamily, and the remaining 12 members clustered with the *A. thaliana* KNOX subfamily, therefore, the 11 TALE gene family members clustered with the *A. thaliana* BELL subfamily were named as the *P. mume* BELL subfamily, and the remaining 12 TALE gene family members were named as the *P.mume* KNOX subfamily (Figure 3).

**Figure 4:**
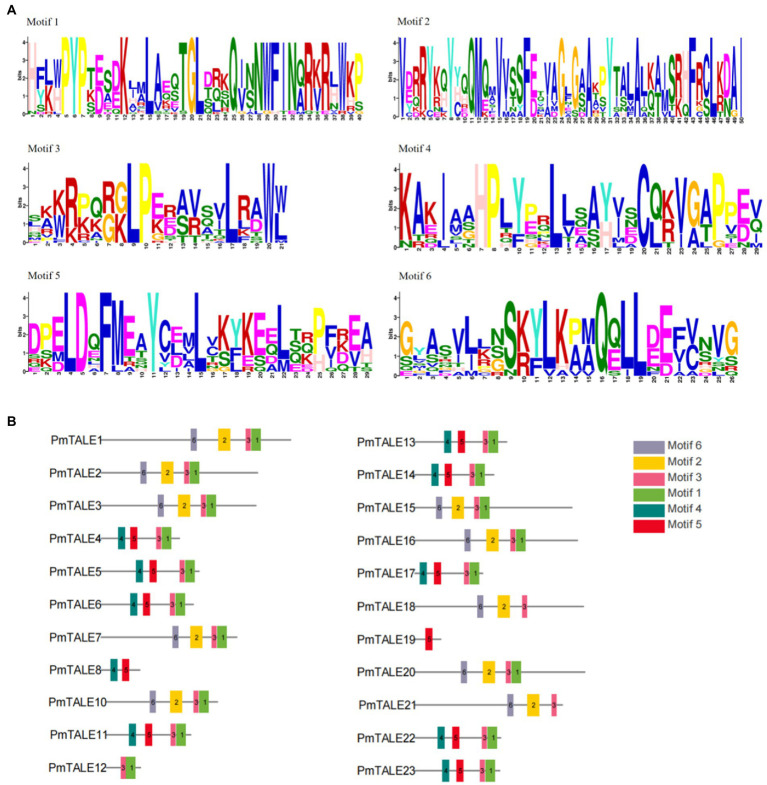
Motif analysis of the protein sequence of the *Prunus mume* TALE gene family, **(A)** shows the sequences of the six kinds of motifs; **(B)** reflects the distribution of the six kinds of motifs on the *P. mume* TALE protein sequences.

We used MEME website to predict amino acid sequences of *P. mume* TALE gene family members and a total of six kinds of motifs were found (Figure 4A). Analysis of the motif contained in all the members of the TALE gene family found that 19 TALE gene family members except PmTALE8, PmTALE18, PmTALE19, and PmTALE21 contained motif1, and 21 TALE gene family members except PmTALE8 and PmTALE19 contained motif3. Analysis of the motif contained in the BELL subfamily found that all the members contained motif2 and motif6. Analysis of the motif contained in the KNOX subfamily found that, except for PmTALE12 and PmTALE19, the rest of the members contained motif4 and motif5, while PmTALE19 only contained motif5, and PmTALE12 contained neither motif4 nor motif5 (Figure 4B).

**Figure 5:**
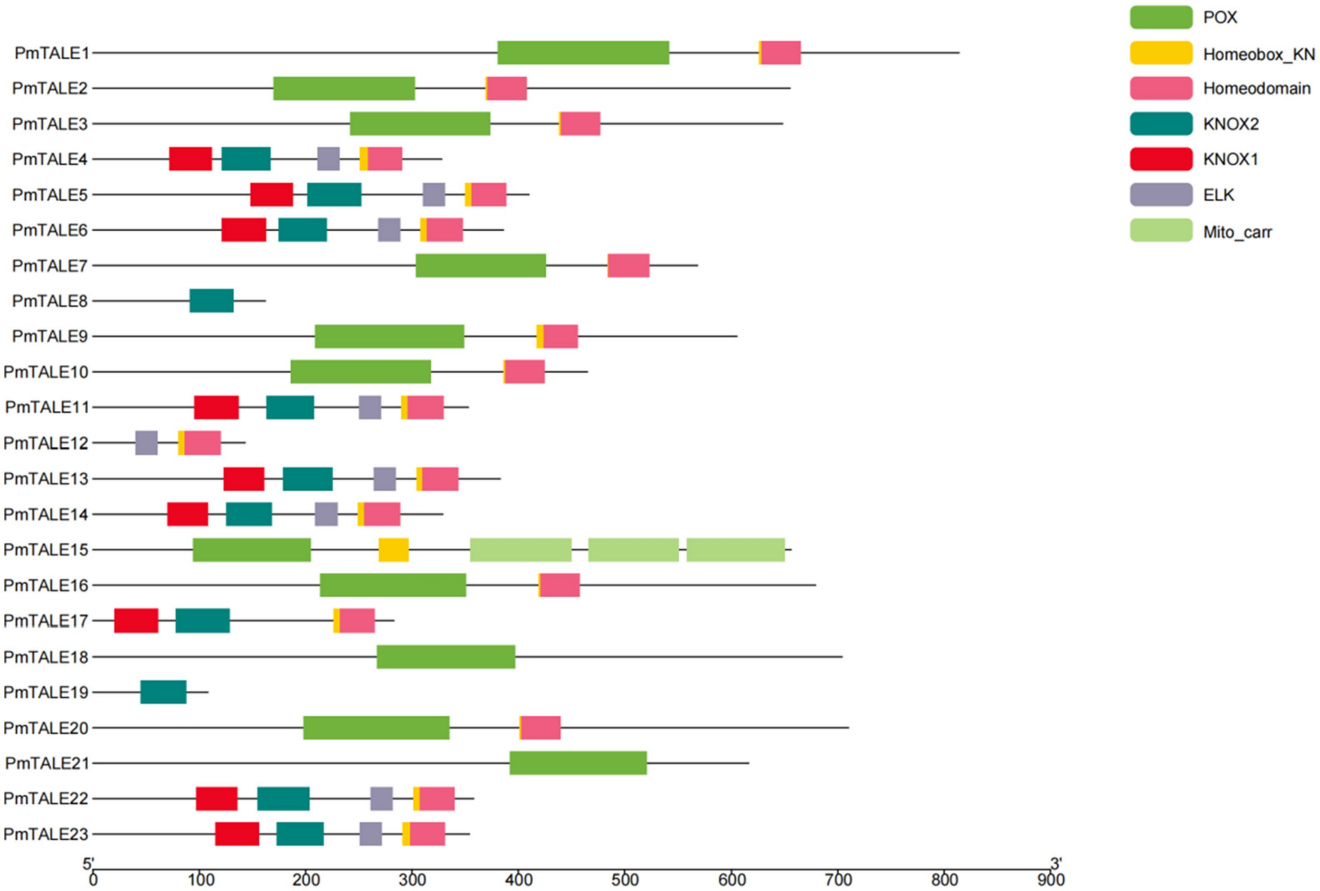
Analysis of the protein sequence domain of the *Prunus mume* TALE gene family.

We used Pfam to align the amino acid sequences of *P. mume* TALE gene family members with known domains, and it was found that the family contained seven domains. Analysis of the domains contained in all members of TALE gene family showed that 19 TALE gene family members except PmTALE8, PmTALE18, PmTALE19, and PmTALE21 contained Homeodomain or Homeobox_KN domain. Analysis of the domains contained in the BELL subfamily found that all members of this subfamily contained POX domains. Analysis of the domains contained in the KNOX subfamily found that, except for PmTALE8, PmTALE12, and PmTALE19, the rest of the members contained KNOX1 and KNOX2 domains, while PmTALE8 and PmTALE19 only contained KNOX2 domains, and PmTALE12 contained neither KNOX1 nor KNOX2 domain. Furthermore, with the exception of PmTALE8, PmTALE17 and PmTALE19, the remaining members of this subfamily contained ELK domain (Figure 5).

Finally, we compared the motif prediction results and the structural domain alignment results, and found that the two results had a corresponding relationship. Motif1 and motif3 roughly correspond to Homeodomain and Homeobox_KN domains, motif2 and motif6 roughly correspond to POX domains, motif4 roughly corresponds to KNOX1, and motif5 roughly corresponds to KNOX2. It is worth mentioning that PmTALE8 and PmTALE19 clustered with the *A. thaliana* KNATM in the phylogenetic tree (Figure 3). The protein length of the *A. thaliana* KNATM is very short, and does not contain the Homeodomain domain of the Homeobox gene family, and only contains the KNOX1 and KNOX2 domains, but it can still play biological functions in the process of plant growth and development (Magnani and Hake, 2008). The domain characteristics of PmTALE8 and PmTALE19 were similar to KNATM, indicating that the predicted protein structure was consistent with the phylogenetic tree clustering results.

### Chromosome location and collinearity analysis of *Prunus mume* TALE gene family

**Figure 6:**
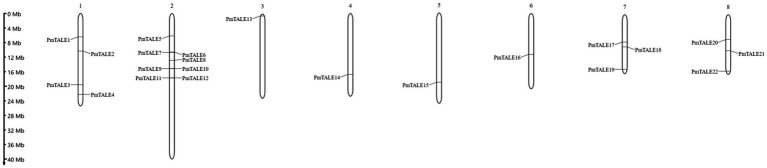
Chromosomal location of the *Prunus mume* TALE gene family.

Chromosome location results showed that except for *PmTALE23* gene, 22 members of the TALE gene family in *P. mume* were all located on chromosomes and were distributed on eight chromosomes. Specifically, chromosome 2 contained eight *TALE* genes. Chromosome 1 contains four *TALE* genes, chromosomes 7 and 8 contain three *TALE* genes, and chromosomes 3–6 contain only one *TALE* gene (Figure 6).

**Figure 7:**
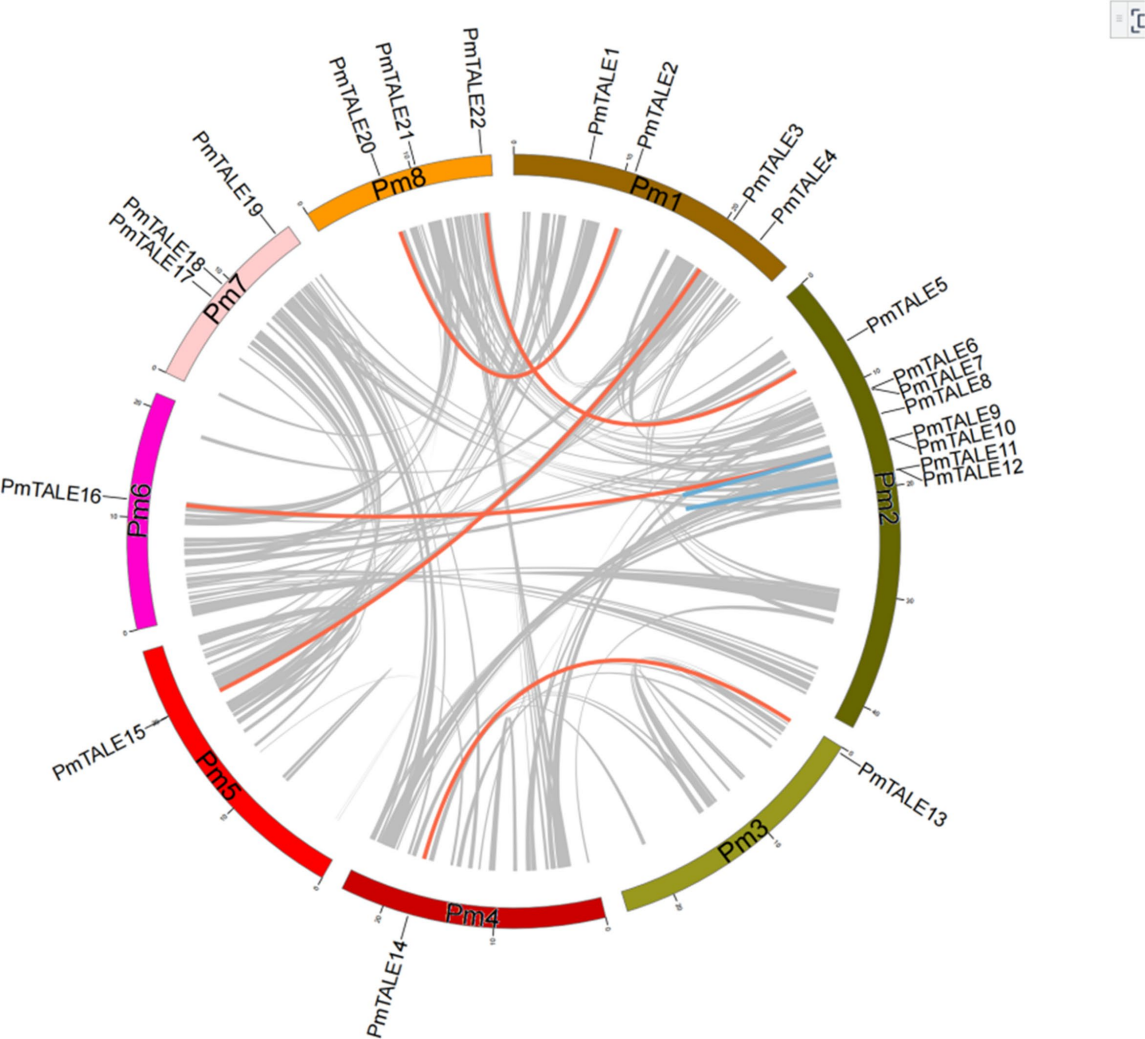
Intra species collinearity analysis of *Prunus mume* TALE gene family. Red lines indicate gene pairs that form genome-wide replication (WGD) or segmental duplications, blue lines indicate gene pairs that form tandem duplications, and grey lines represent other collinear gene pairs of non-TALE gene family members within the genome.

Intra-species collinearity analysis was performed on 22 members of the TALE gene family located on chromosomes and the results showed that a total of 13 members participated in the formation of seven collinear pairs, as follows: *PmTALE2* and *PmTALE20*, *PmTALE3* and *PmTALE15*, *PmTALE5* and *PmTALE22*, *PmTALE9* and *PmTALE16*, *PmTALE13* and *PmTALE14*, *PmTALE9* and *PmTALE10*, and *PmTALE11* and *PmTALE12*. Among them, the first five pairs were genome-wide replication (WGD) or fragment replication, and the last two pairs were tandem replication. It indicated that fragment duplication and tandem duplication events were the main reasons for the expansion of members of the TALE gene family in *P. mume* (Figure 7).

The Ka to Ks ratio was calculated for collinear pairs with WGD or fragment duplication and tandem duplication to predict the adaptive evolution of gene CDS regions. The results showed that the ratios of Ka to Ks for all collinear pairs ranged from 0.02 to 0.67, which were all less than 1, indicating that these genes were selected by different degrees of purification. In addition, the evolutionary time of the five collinear pairs with WGD or fragment duplication was relatively close, which was estimated to be 89.2–95.7 MY. However, the evolutionary time of the two collinear pairs with tandem duplication was far apart, specifically, *PmTALE9* and *PmTALE10* evolved as early as about 1485.3 MY, while *PmTALE11* and *PmTALE12* evolved at about 5.5 MY (Table 2).

**Table 2:** Ka/Ks value calculation and evolution time prediction of collinear pairs within the *Prunus mume* TALE gene family.

Collinear pairs		Ka	Ks	Ka/Ks	Date (MY)	Duplication type
*PmTALE2*	*PmTALE20*	0.31	1.74	0.18	95.67	WGD or segmental duplications
*PmTALE3*	*PmTALE15*	0.49	1.66	0.30	91.30	WGD or segmental duplications
*PmTALE5*	*PmTALE22*	0.15	1.62	0.09	89.20	WGD or segmental duplications
*PmTALE9*	*PmTALE16*	0.37	1.71	0.22	93.83	WGD or segmental duplications
*PmTALE9*	*PmTALE10*	0.56	27.03	0.02	1485.32	Tandem duplication
*PmTALE11*	*PmTALE12*	0.07	0.10	0.67	5.45	Tandem duplication
*PmTALE13*	*PmTALE14*	0.24	1.63	0.14	89.51	WGD or segmental duplications

In order to preliminarily explore the evolutionary relationship of TALE gene family between *P. mume* and other *Prunus* species, we performed the inter-species collinearity analysis of the 22 members of this family located on chromosomes. The other four *Prunus* species selected for comparative analysis were *P. armeniaca*, *P. persica*, *P. avium*, and *P. dulcis*. China is the main origin of *P. armeniaca*, while *P. persica* has been cultivated in China as early as 2,000 BC. Different from the first two kinds of plants, the latter two kinds of plants are mainly distributed in foreign countries. Among them, *P. avium* is a cherry native to Europe, western Turkey, northwestern Africa, and western Asia. *P. dulcis* is native to Israel, west Jordan, Lebanon, south Turkey, Turkmenistan, and Uzbekistan.

**Figure 8:**
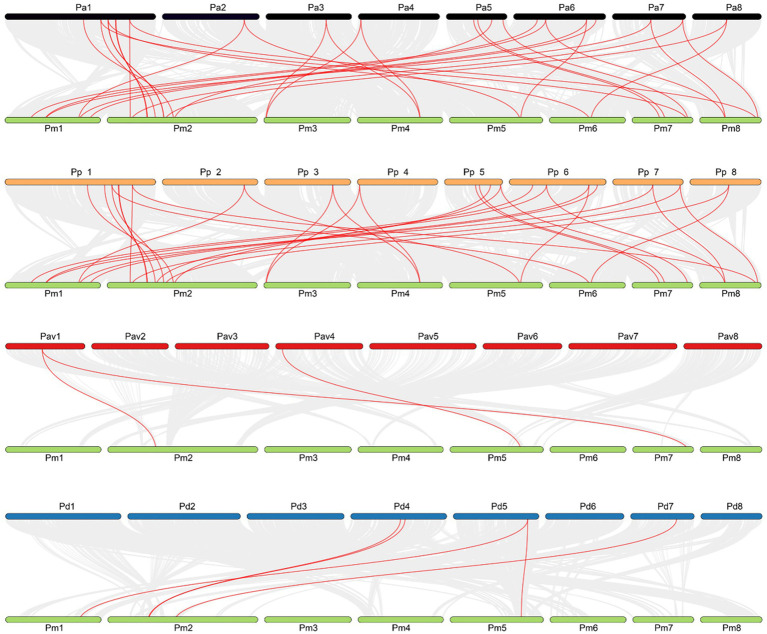
Collinear analysis of TALE gene family between *Prunus mume* and four kinds of *Prunus* species. The red lines represent gene pairs with a collinear relationship, grey lines represent other collinear gene pairs of non-TALE gene family members across genomes; Green represents *P. mume* chromosomes, black represents *P. armeniaca* chromosomes, orange represents *P. persica* chromosomes, red represents *P. avium* chromosomes, and blue represents *P. dulcis* chromosomes.

The results of inter-species collinearity analysis showed that genes with collinear relationship with members of the *P. mume* TALE gene family could be found in other four *Prunus* species. Specifically, a total of 24 genes on the *P. armeniaca* chromosome formed 32 collinear gene pairs with *P. mume* *TALE* genes. These 24 genes were distributed on eight chromosomes of *P. armeniaca.* Among them, chromosome 1 contained the largest number of genes, a total of 7, while chromosomes 3, 4, and 8 contained only one gene. A total of 22 genes on the *P. persica* chromosome formed 31 collinear gene pairs with the *P. mume* *TALE* genes. These 22 genes were distributed on eight chromosomes of *P. persica.* Among them, chromosome 1 contained the largest number of genes, a total of 6, while chromosomes 3, 4, and 8 contained only one gene. Overall, the results of the collinearity analysis of TALE gene family between *P. mume* with *P. armeniaca* are similar to that between *P. mume* with *P. persica*. On the chromosome of *P. dulcis*, only five genes formed five collinear gene pairs with members of the *P. mume* TALE gene family, and these five genes were distributed on chromosomes 4, 5, and 7. On the *P. avium* chromosome, there were only two genes that formed three collinear gene pairs with the *P. mume* *TALE* genes, and these two genes were distributed on chromosomes 1 and 4. Overall, compared with *P. armeniaca* and *P. persica*, the number of collinear gene pairs of *TALE* genes formed between the *P. mume* with *P. dulcis* or *P. avium* was very small. To some extent, this indicates that *P. mume* is closely related to *P. armeniaca* and *P. persisca*, but far away from *P. dulcis* and *P. avium*. The origins of *P. mume*, *P. armeniaca* and *P. persica* are mainly distributed in China, while the origins of *P. dulcis* and *P. avium* are mainly distributed in foreign countries, indicating that the distance of origin of these five *Prunus* species is consistent with the distance of collinear relationship (Figure 8).

### Prediction of protein interactions and promoter elements of *Prunus mume* TALE gene family members

**Figure 9:**
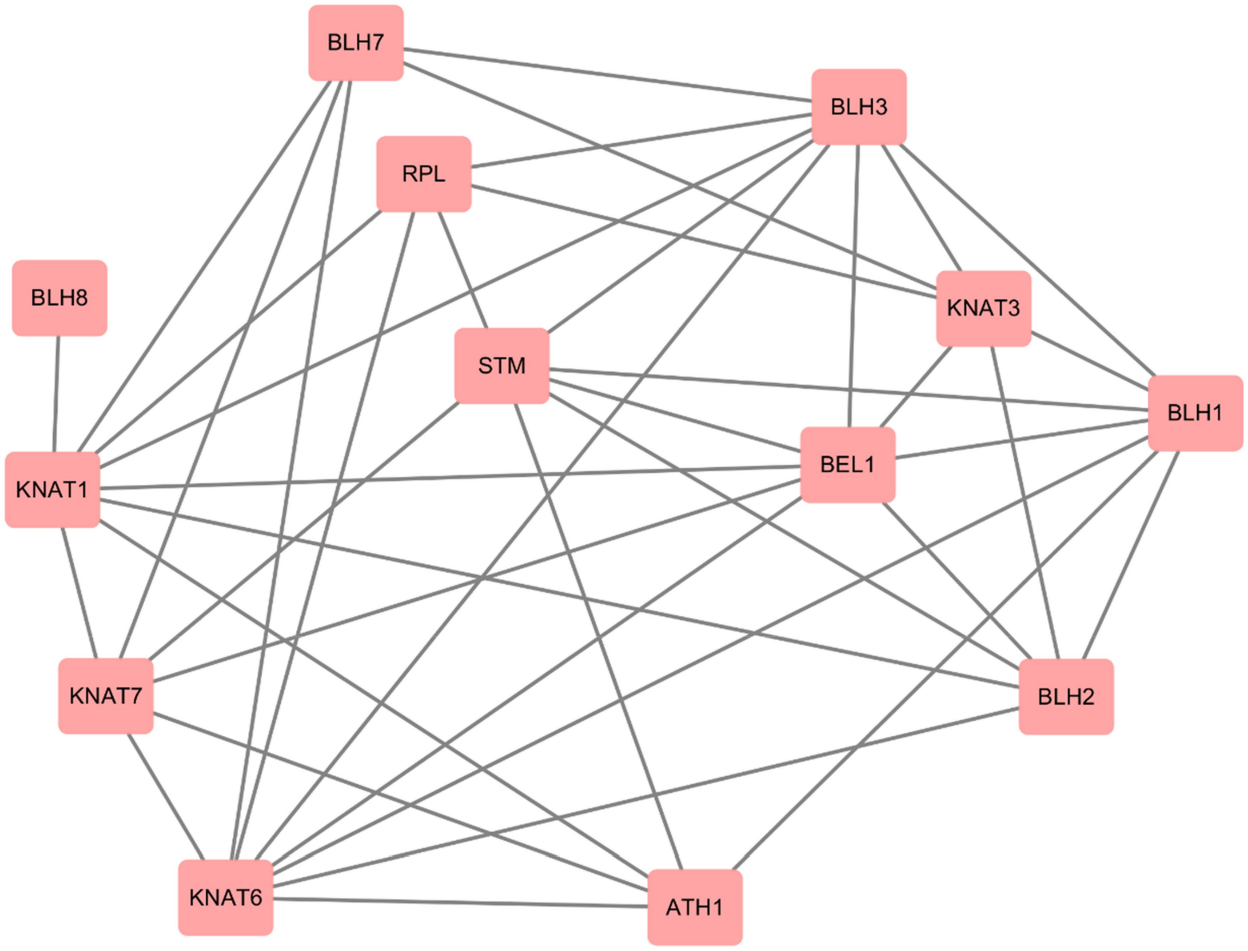
Prediction of protein interactions within *Prunus mume* TALE gene family. STM: PmTALE13, PmTALE14; KNAT1: PmTALE6; KNAT3: PmTALE5, PmTALE22; KNAT6: PmTALE4, PmTALE11, PmTALE12, PmTALE23; KNAT7: PmTALE17; RPL: PmTALE3, PmTALE15; ATH1: PmTALE7; BEL1: PmTALE18; BLH1: PmTALE2, PmTALE20; BLH2: PmTALE21; BLH3: PmTALE9; BLH7: PmTALE16; BLH8: PmTALE1.

We used STRING website to predict the protein interactions among members of *P. mume* TALE gene family, and the results showed that there were many interactions among members of this family. First, there are four types of protein interactions within the KNOX subfamily, specifically, STM (PmTALE13, PmTALE14) and KNAT1 (PmTALE6), STM (PmTALE13, PmTALE14) and KNAT7 (PmTALE17), KNAT6 (PmTALE4, PmTALE11, PmTALE12, and PmTALE23) and KNAT7 (PmTALE17), KNAT7 (PmTALE17) and KNAT1 (PmTALE6). In addition, there are also eight kinds of protein interactions within the BELL subfamily, including ATH1 (PmTALE7) and BLH1 (PmTALE2, PmTALE20), BLH1 (PmTALE2, PmTALE20) and BEL1 (PmTALE18), BLH1 (PmTALE2, PmTALE20) and BLH2 (PmTALE21), BLH1 (PmTALE2, PmTALE20) and BLH3 (PmTALE9), BLH2 (PmTALE21) and BEL1 (PmTALE18), BLH3 (PmTALE9) and BLH7 (PmTALE16), BLH3 (PmTALE9) and RPL (PmTALE3, PmTALE15), and BEL1 (PmTALE18) and BLH3 (PmTALE9). Moreover, there are also interactions between the KNOX subfamily and the BELL subfamily, and the number is large, with a total of 29 types. For example, STM (PmTALE13, PmTALE14) and ATH1 (PmTALE7), KNAT3 (PmTALE5, PmTALE22) and RPL (PmTALE3, PmTALE15), and so on. In summary, the members of the *P. mume* TALE gene family may form dimers or multimers through protein interactions to perform transcriptional regulatory functions (Figure 9).

**Figure 10:**
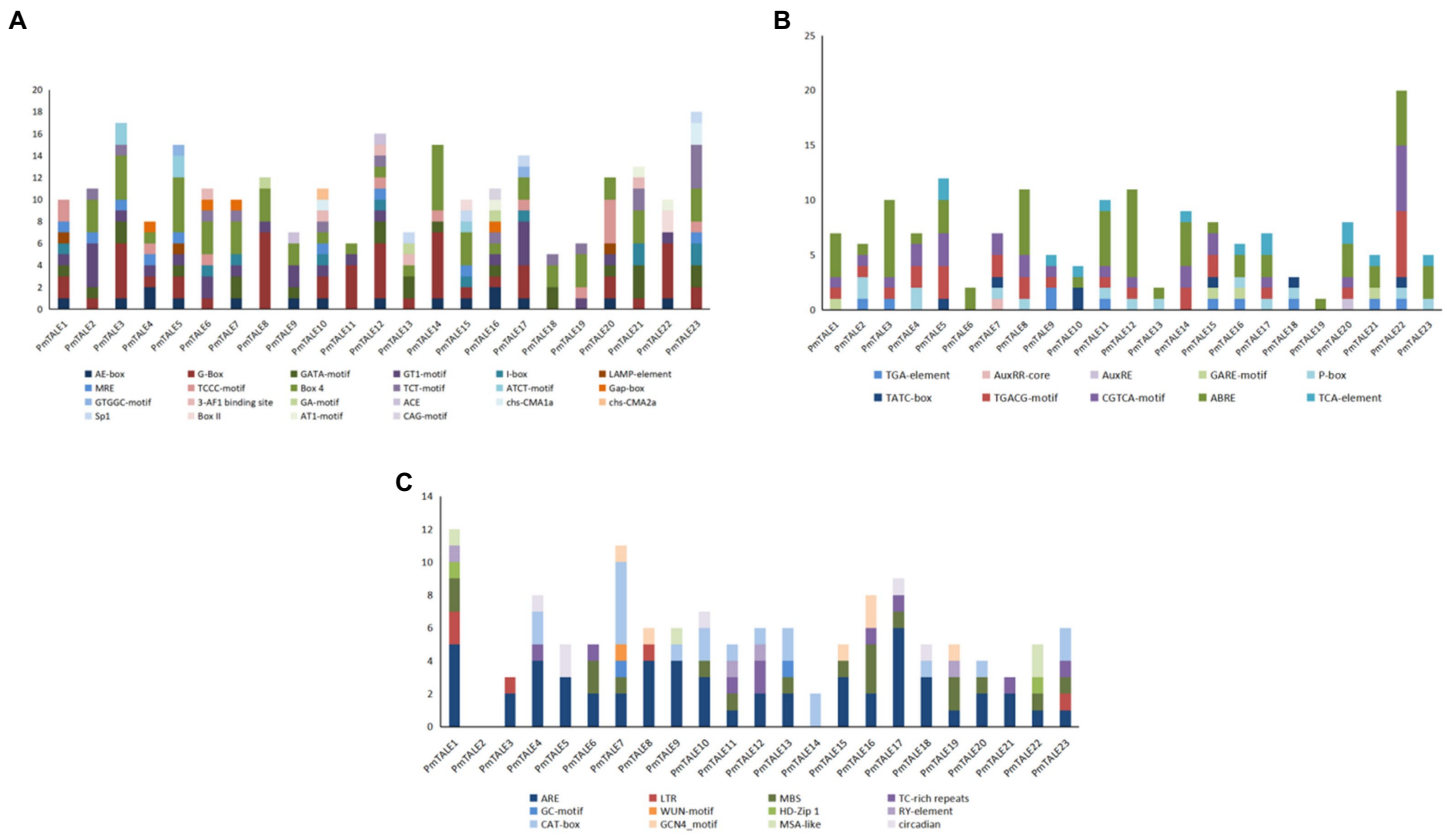
Cis element analysis of promoter of *Prunus mume* TALE gene family members. **(A)** shows the types and numbers of light-responsive elements contained in the *TALE* gene promoter; **(B)** shows the types and numbers of hormone response elements contained in the *TALE* gene promoter; and **(C)** shows the types and numbers of other elements contained in the *TALE* gene promoter. The ordinate represents the number of elements.

Promoter analysis results showed that there were 22 light-responsive elements in the promoters of the *P. mume* TALE gene family members. Among them, the *PmTALE12* promoter contained the most types of light-responsive elements, with a total of 11 types, while the *PmTALE11* and *PmTALE18* promoters contained the least types of light-responsive elements, three types. The *PmTALE23* promoter contained the largest number of light-responsive elements, with a total of 18, and the *PmTALE18* promoter contained the least number of light-responsive elements, only 6 (Figure 10A). In addition, there were 10 types of hormone response elements in the promoters of the *P. mume* TALE gene family members, including three kinds of auxin response elements, three kinds of gibberellin response elements, two kinds of methyl jasmonate response elements, one kind of abscisic acid response element, and one kind of salicylic acid response element. Among them, the *PmTALE22* promoter contained the largest number of hormone response elements, with a total of 20, and the *PmTALE19* promoter contained the least number of hormone response elements, only one (Figure 10B). Moreover, the promoters of the *P. mume* TALE gene family members also contained regulatory elements that respond to abiotic stress and plant development. Specifically, abiotic stress includes low temperature, drought, hypoxia induction, trauma response, etc., and plant development includes palisade mesophyll cell differentiation, meristem development, endosperm development, cell cycle, circadian rhythm, etc. (Figure 10C). In conclusion, the *P. mume* TALE gene family may be regulated by light signals and various hormonal signals, participate in the development of *P. mume* and respond to various abiotic stresses.

### Expression profile of *Prunus mume* TALE gene family members in different tissues and different stem developmental stages

**Figure 11:**
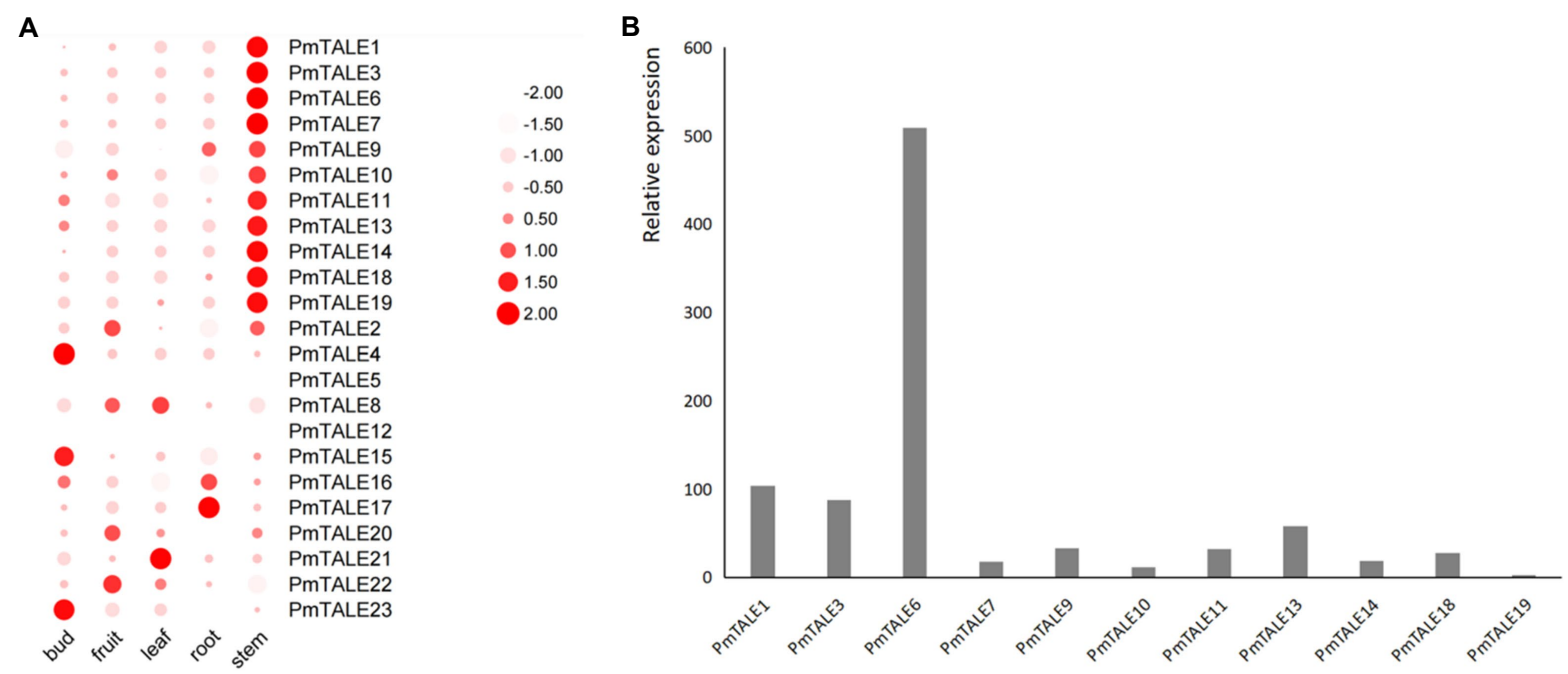
Expression profiling of the *Prunus mume* TALE gene family based on RNA sequencing results. **(A)** The heatmap parameters are set to: row scale and scale size by area; **(B)** Relative expression levels of some *TALE* genes in stem. The abscissa represents the 11 *TALE* genes that are specifically highly expressed in the stem, and the ordinate represents the relative expression of genes in the stem.

The expression profiles of *P. mume* TALE gene family members in five different tissues showed significant differences in the expression distribution of members. The first 11 members were highly expressed in the stem (Figure 11A). Among these 11 members, *PmTALE6* has the highest expression, while *PmTALE19* has the lowest expression in stem (Figure 11B). In addition, *PmTALE4*, *PmTALE15,* and *PmTALE23* were highly expressed in flower buds, *PmTALE21* and *PmTALE8* were highly expressed in leaves, *PmTALE16* and *PmTALE17* were significantly expressed in roots, and *PmTALE2*, *PmTALE20,* and *PmTALE22* showed high expression in fruits (Figure 11A).

**Figure 12:**
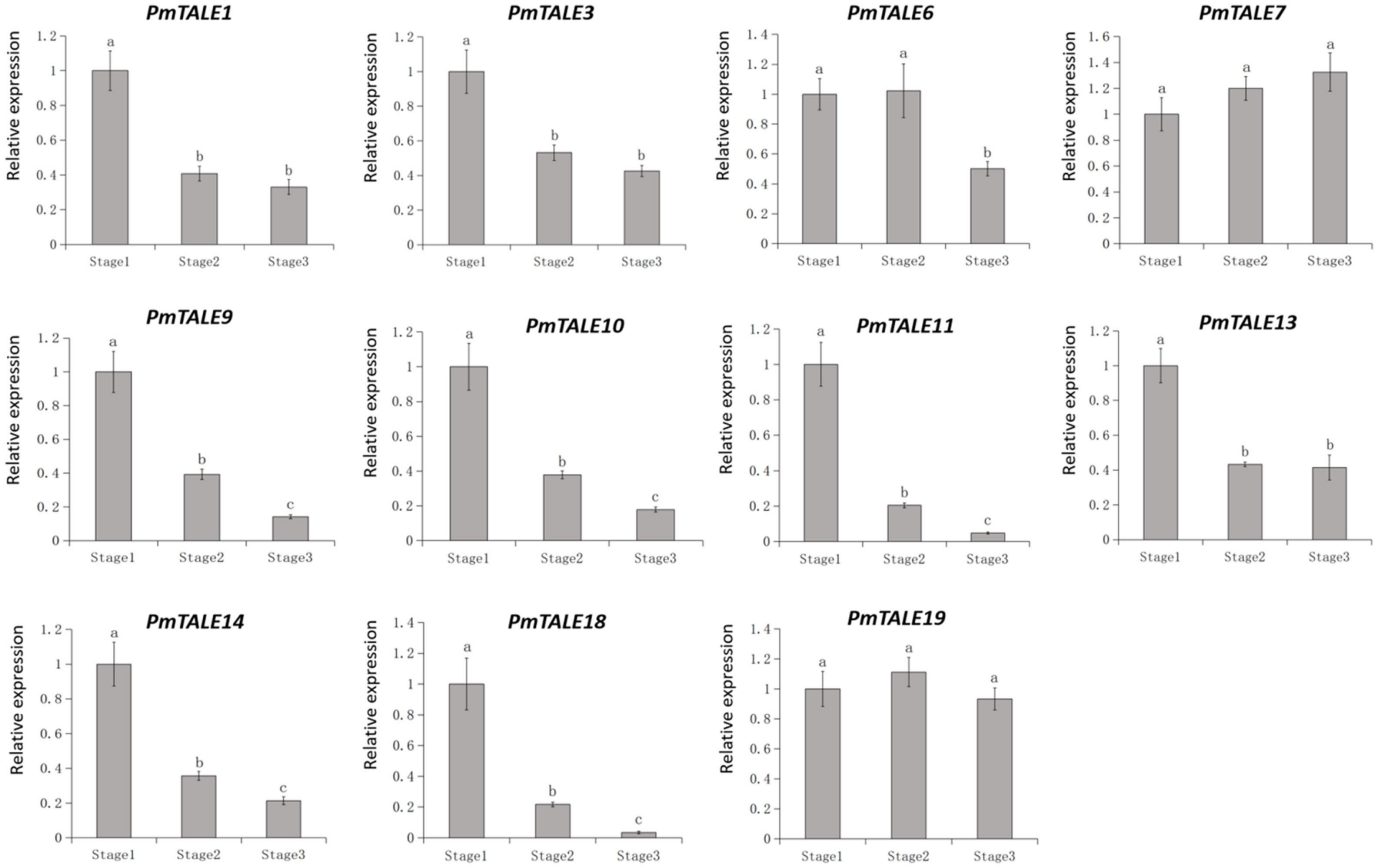
The expression profile of *Prunus mume* TALE gene family at different developmental stages of the stem. The abscissa represents the three stages of *Prunus mume* stem development, and the ordinate represents the relative expression level.

Previous reports have shown that *TALE* genes are involved in stem development of plants (Testone et al., 2012; Zhao et al., 2019; Que et al., 2022). In this study, in order to preliminarily explore the effect of *TALE* genes on *P. mume* stem development, we selected 11 members highly expressed in the *P. mume* stem to perform further expression pattern analysis in three different developmental stages of stem. According to the level of expression and the significance of the difference, the expression patterns can be divided into four types (Figure 12). The first type included *PmTALE7* and *PmTALE19*, and there was no significant difference in the expression levels among the three developmental stages. The second type only included *PmTALE6*, and the expression level in the third stage was significantly lower than that in the first and second stages, but there was no significant difference between the first and second stages. The third type included *PmTALE1*, *PmTALE3*, and *PmTALE13*, and the expression levels in the first stage were significantly higher than that in the second and third stages, but there was no significant difference in the expression levels between the second and third stages. The fourth type included *PmTALE9*, *PmTALE10*, *PmTALE11*, *PmTALE14*, and *PmTALE18*, and the expression levels decreased gradually from the first stage to the third stage, and there were significant differences between each stage. Overall, all nine members except *PmTALE7* and *PmTALE19* were highly expressed in the first stage of stem development. Therefore, it is speculated that the highly expressed *TALE* genes in stem may be involved in regulating the early development of *P. mume* stem.

### Expression profile of *Prunus mume* TALE gene family members under different hormone treatments and different abiotic stress conditions

**Figure 13:**
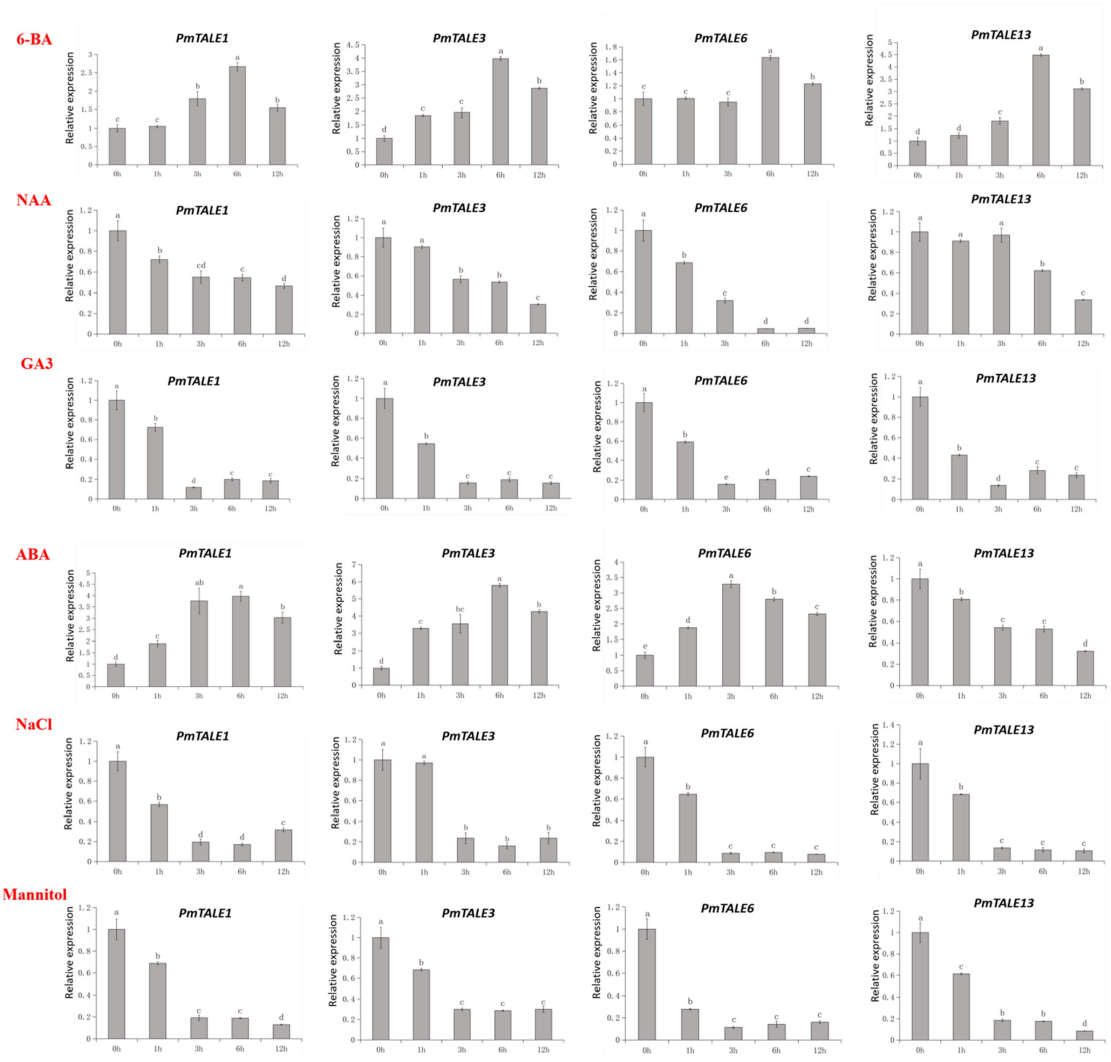
Expression profiling of *Prunus mume* *TALE* genes under different hormone treatments and different abiotic stress conditions. The abscissa represents the treatment time of hormone or abiotic stress, and the ordinate represents the relative expression level.

Based on the above studies, nine of the 11 highly expressed *TALE* genes in *P. mume* stem showed high expression in the early stage of stem development. Among these nine genes, *PmTALE1*, *PmTALE3*, *PmTALE6*, and *PmTALE13* had the highest expression levels (Figure 11B). Therefore, we selected these genes for further study to explore their responses against four kinds of hormone treatments and two kinds of abiotic stress treatments during the early development of *P. mume* stem. 6-BA treatment upregulated the expression of these four *TALE* genes, *PmTALE3* was significantly upregulated at 1 h, *PmTALE1* and *PmTALE13* were significantly upregulated at 3 h, and *PmTALE6* was significantly upregulated at 6 h. NAA treatment downregulated the expression of these four *TALE* genes, *PmTALE1* and *PmTALE6* were significantly down-regulated at 1 h, *PmTALE3* was significantly downregulated at 3 h, and *PmTALE13* was significantly downregulated at 6 h. GA3 treatment also downregulated the expression of these four *TALE* genes, and all of them were significantly downregulated at 1 h. The expression of *PmTALE1*, *PmTALE3*, and *PmTALE6* were all upregulated under ABA treatment, while the expression of *PmTALE13* was downregulated, and their expression levels at 1 h were significantly different from those at 0 h. NaCl treatment downregulated the expression of these four genes, *PmTALE1*, *PmTALE6*, and *PmTALE13* were significantly downregulated at 1 h, and *PmTALE3* was significantly downregulated at 3 h. After mannitol treatment, the expression of four genes was significantly down-regulated at 1 h. In conclusion, in the early stage of *P. mume* stem development, four *TALE* genes responded to all hormone treatments, specifically, 6-BA promoted the expression, NAA and GA3 inhibited the expression, while the effects of ABA on *TALE* genes were not consistent. In addition, the four *TALE* genes were all downregulated under salt stress and drought stress, which may be due to the poor resistance of the *P. mume* young stem to external stress in the early developmental stage or the downregulated expression of these genes may help to improve the resistance of *P. mume* (Figure 13).

## Discussion

As early as 3,000 years ago, *P. mume* has been domesticated as an ornamental plant in China, and the fruit is edible. In addition, *P. mume* is also one of the earliest *Prunus* plants to have their genome sequenced (Zhang et al., 2012). In this study, a total of 23 *TALE* genes are identified based on the *P. mume* genome, which is close to the number of *TALE* genes (22) in *A. thaliana* and *P. armeniaca*. However, the number of *TALE* genes (36) in *P. persica*, which is also a *Prunus* plant, is relatively large. There are also differences in the number of *TALE* genes found by researchers in other species. For example, *Punica granatum* contains only 17 members (Wang et al., 2020), radish, walnut, and poplar contain 33, 30, and 35 members, respectively (Zhao et al., 2019, 2022; Guo et al., 2022), and *G. hirsutum* contains as many as 89 members (Razzaq et al., 2020). The phylogenetic tree results showed that, similar to *A. thaliana*, the TALE proteins of *P. mume* and two other *Prunus* species (*P. armeniaca* and *P. persica*) could all be divided into two subfamilies, KNOX and TELL subfamilies, respectively, and the number of members of the two subfamilies is not much different. In addition, the KNOX subfamilies of the above three *Prunus* species all had KNATM branch. It is worth mentioning that this branch is only present in eudicots, for example, *Theobroma cacao*, *Populus trichocarpa*, and *Gossypium hirsutum* all contain KNATM like proteins (Zhang et al., 2021b), while no member of KNATM like proteins has been identified in the KNOX proteins of orchids (Zhang et al., 2022a). The domain analysis of *P. mume* TALE proteins showed that almost all members contained Homebox_KN domain, all members of BELL subfamily contained POX domain, and most members of KNOX subfamily contained KNOX1, KNOX2 and ELK domain. Previous studies have shown that the Homebox_KN domain is located at the C-terminal of the protein and is related to the function of DNA binding, while ELK domain can be used as a nuclear localization signal and is related to transcriptional inhibition (Kerstetter et al., 1994; Scofield and Murray, 2006).

In order to further analyze the evolutionary relationship within the TALE gene family of *P. mume*, we conducted an intra-species collinearity analysis and found a total of seven pairs of collinearity, of which four pairs existed in the BELL subfamily, and the other three pairs existed in the KNOX subfamily. The seven collinear pairs included four fragment duplications and two tandem duplication events, leading to the expansion of the TALE gene family in *P. mume*. In addition, the Ka/Ks values of the collinear pairs were all less than 1, indicating that these gene pairs were all selected by different degrees of purification. Studies have shown that evolution caused by gene duplication events is prevalent in angiosperms. For example, there is obvious expansion phenomenon in woody bamboo TALE gene family, which is caused by many duplication events (Que et al., 2022). Whole-genome duplication events occurred in *G. raimondii* and *G. arboretum* (Huang et al., 2020), and there were 34 gene pairs with fragment duplication in the *TALE* genes of *G. hirsutum* obtained by crossing the above two species, and also selected by purification (Zhang et al., 2021b). In addition, interspecies collinearity analysis can be used to study the evolution of gene families among species. For example, Que. et al. found that the *TALE* genes of Moso Bamboo and the *TALE* genes of rice have a high collinearity relationship (Que et al., 2022). In this study, we used the inter-species collinearity analysis to study the collinearity of *TALE* genes between *P. mume* and four other *Prunus* species. The origin of *P. mume*, *P. armeniaca*, and *P. persica* are mainly distributed in China, and the results show that their collinearity is also closer. At the same time, the phylogenetic tree analysis mentioned above also showed that the overall evolutionary pattern of TALE gene family of *P. armeniaca* and *P. persica* is similar to *P. mume*. While the origin of *P. dulcis* and *P. avium* are mainly distributed abroad, and the analysis results show that the collinear relationship between *P. mume* and these two species of *Prunus* is relatively far. Therefore, it can be seen that the distance of the collinearity is consistent with the distance of the origin.

The Homebox_KN domain and ELK domain of the TALE gene family are both related to transcriptional regulation, and as a transcription factor, this family plays an important role in regulating plant growth and development. In recent years, there have been many reports on the function of the *TALE* genes. For example, Wang et al. predicted that the *TALE* genes of pomegranate may have regulatory effects on SAM, flower, and ovule development (Wang et al., 2020). The expression levels of *TALE* genes in walnut were different at different stages of flower bud development (Guo et al., 2022). Downregulated expression of cotton TALE gene family member *GhSTM3* affects flowering time (Zhang et al., 2021b). During the rapid growth period of *Phyllostachys edulis*, members of the TALE gene family may affect the formation of secondary cell walls in internodes by regulating the synthesis of xylan (Que et al., 2022). Nearly half of (17) *TALE* genes in poplar are highly expressed in stem, which may play a key regulatory role in wood formation (Zhao et al., 2019). Similar to the results of poplar, in this study, we also found nearly half of (11) *TALE* genes that were specifically highly expressed in *P. mume* stem based on the expression profiles of different tissues, indicating that *P. mume* *TALE* genes may have an important regulatory role in stem development. In addition, although the expression levels of these genes were different in different developmental stages of stem, they were mainly highly expressed in the early developmental stage of stem, further indicating that *TALE* genes may mainly play a role in the early developmental process of stem. Similarly, it has been previously reported that *KNOPE1*, a member of the peach TALE gene family, is also involved in early stem development and is expressed in cortex and procambium, preventing lignification of primary stem by inhibiting lignin-related genes (Testone et al., 2012). However, considering that some *TALE* genes of *P. mume* are not specifically highly expressed in stem, but their expression levels are not low in stem, they may also have important functions in stem development and are worthy of further research in the future. In addition, TALE proteins often form dimers to function, for example, yeast two-hybrid experiments found that different poplar TALE proteins can form heterodimers (Zhao et al., 2019). Similarly, this study also predicted that heterodimers may be formed within the *P. mume* KNOX and BELL subfamilies and between the two subfamilies. Therefore, the TALE proteins of *P. mume* may affect the early development of stem through the formation of heterodimers.

The effects of transcription factors on growth and development are usually closely related to hormonal pathways, and previous studies have found that the function of the *TALE* genes is related to the hormone pathway of plants. For example, the promoter region of the orchid *KNOX* genes was enriched with MeJA and ABA-responsive cis-elements (Zhang et al., 2022a), the promoter sequence of pomegranate *TALE* genes contained auxin and gibberellin-responsive cis-elements (Wang et al., 2020), and the expression of the *KNOX* genes of *Caucasian clover* could respond to changes in 6-BA, IAA, and KT signals from external application (Zhang et al., 2022b). Infection of *A. thaliana* leaves by *R. fascians* can cause local CK responses and reduce GA signaling, which may provide a suitable environment for the expression of *KNOX* genes, resulting in the formation of leaf edge serration (Depuydt et al., 2008). *MdKNOX19*, a member of the apple KNOX subfamily, is an ABA-responsive gene and overexpression of *MdKNOX19* increases the ABA sensitivity of apple callus (Jia et al., 2021). In this study, the 2000 bp promoter of the *P. mume* *TALE* genes was analyzed, and a variety of hormone response elements were also found, including auxin, gibberellin, methyl jasmonate, abscisic acid and salicylic acid. At the same time, the exogenous hormone application experiment was used to explore the response of the top four *TALE* genes in *P. mume* stem expression (*PmTALE1, PmTALE3, PmTALE6,* and *PmTALE13*) to four kinds of hormones during the early stage of stem development, and it was found that the expression of these four genes could all respond to the changes of external hormones. The 6-BA upregulated their expression, NAA and GA3 downregulated their expression, and ABA had inconsistent effects on them, showing the complexity of hormone action, and further indicating that hormones may affect early development of *P. mume* stem by regulating the expression of *TALE* genes.

There is a correlation between hormone response and plant resistance to abiotic stress. For example, ethylene-related gene expression models suggest that ethylene may indirectly participate in the induction of dormancy, thereby enhancing cold/freezing tolerance of *P. mume* (Li et al., 2021). *Populus alba × P. glandulosa* KNOX subfamily member *PagKNAT2/6b* can mediate drought response by down-regulating *PagGA20ox1* gene of GA pathway (Song et al., 2021). Similarly, the expression of *TALE* genes is not only regulated by hormones, but also affected by abiotic stress (Tsuda and Hake, 2015; Niu and Fu, 2022). The promoters of TALE gene family members in soybean contained cis-elements that responded to various stresses, and the expression level of *GmTALE* gene could change in response to salt stress and drought stress (Wang et al., 2021). Eleven members of poplar *TALE* genes respond to salt stress (Zhao et al., 2019). A variety of *TALE* genes in cotton are upregulated under various abiotic stresses, and may play a role in coping with stressful environments (Razzaq et al., 2020). A later study found that the silencing of *GhKNOX10* and *GhKNOX14* in cotton reduced the tolerance of seedlings to salt stress, while the silencing of *GhKNOX2* enhanced the salt tolerance of cotton seedlings (Zhang et al., 2021b). Members of the pear KNOX subfamily can respond to drought stress treatment, specifically, the expression of *PbKNOX7/13* is increased under drought stress, while the expression of *PbKNOX5/16* is inhibited under drought stress (Liu et al., 2022). In this study, the promoter analysis of *TALE* genes of *P. mume* revealed a variety of abiotic stress elements, including low temperature, drought, hypoxia induction, and trauma response. Then, the responses of the top four *TALE* genes in *P. mume* stem expression (*PmTALE1, PmTALE3, PmTALE6,* and *PmTALE13*) to drought stress and salt stress during the early stage of stem development were investigated. The results showed that these four genes were all downregulated under stress conditions, which may be due to the poor resistance of the *P. mume* young stem to external stress in the early developmental stage. Alternatively, the downregulated expression of these genes may help to improve the resistance of *P. mume,* which requires further research in the future.

## Conclusion

We identified a total of 23 *TALE* genes in *P. mume*. Phylogenetic tree showed that TALE proteins were divided into KONX subfamily and BELL subfamily. The results of protein interaction prediction showed that a variety of heterodimers could be formed between TALE proteins. Intra species collinearity analysis showed that fragment replication and tandem replication events were the main reasons for the expansion of the TALE gene family members and the collinearity analysis between species showed that the collinearity of *TALE* genes between *P. mume* and the other four *prunus* species was consistent with the distance of origin. Eleven members of the *P. mume* *TALE* genes were specifically highly expressed in the stem, mainly in the early stage of stem development. Cis element analysis showed that the promoter of *P. mume* *TALE* genes contained a variety of hormones and abiotic stress response elements, and *TALE* genes could respond to hormone or abiotic stress treatments during the early stage of stem development.

## Data availability statement

The original contributions presented in the study are included in the article/Supplementary Material, further inquiries can be directed to the corresponding author/s.

## Author contributions

QY, CY, and QZ conceived and designed the experiments. QY performed the experiments and analyzed the data. TC carried out some of the experiments. JW supervised the conduct of the experiment. QY prepared the original draft. CY and QZ reviewed and edited the manuscript. All authors contributed to the article and approved the submitted version.

## Funding

This research was funded by the National Key R & D Program of China (2019YFD1001500) and Special Fund for Beijing Common Construction Project.

## Conflict of interest

The authors declare that the research was conducted in the absence of any commercial or financial relationships that could be construed as a potential conflict of interest.

## Publisher’s note

All claims expressed in this article are solely those of the authors and do not necessarily represent those of their affiliated organizations, or those of the publisher, the editors and the reviewers. Any product that may be evaluated in this article, or claim that may be made by its manufacturer, is not guaranteed or endorsed by the publisher.

## Footnotes

^1^ https://www.ncbi.nlm.nih.gov

^2^ https://services.healthtech.dtu.dk/service.php?TMHMM-2.0

^3^ http://mg2c.iask.in/mg2c_v2.1

^4^ http://plants.ensembl.org/index.html
