# Supplementary material for: Genome-wide identification of three-amino-acid-loop-extension gene family and their expression profile under hormone and abiotic stress treatments during stem development of Prunus mume
Source: Front Plant Sci. 2022 Sep 23;13:1006360. doi: 10.3389/fpls.2022.1006360 (PMC9538144; doi:10.3389/fpls.2022.1006360)
Supplement: Supplementary file 1 [file Data_Sheet_1.docx]

Supplementary Material

# Supplementary Table

**Table S1.** Primer Sequences of qPCR

| **Primer name** | **Primer sequence** |
| --- | --- |
| *PmTALE1*-F | CGGTTGCCGGTCTAAGTTCT |
| *PmTALE1*-R | TCCGTATGTGCTTGAGCTGG |
| *PmTALE3*-F | GAGAGCTTCAGCCCAACTCA |
| *PmTALE3*-R | AAACCTCGTCGAGCATGGAA |
| *PmTALE6*-F | TTCCTGTACGCCTCACCAAA |
| *PmTALE6*-R | GTGAAAATCACCACCACCGC |
| *PmTALE7*-F | CCCCCACTGAGAGTGAAAGG |
| *PmTALE7*-R | TTTCAGGCAAGCCTCTCTGG |
| *PmTALE9*-F | TGCGTGATGCAATCAAAGGC |
| *PmTALE9*-R | GTAGCGAAGGCGGGGTATTA |
| *PmTALE10*-F | CTCCATTGCTGCCCAAAACC |
| *PmTALE10*-R | GGCAGGACACCAAGAGACTG |
| *PmTALE11*-F | AAGCCAAAATTGCCTCGCAC |
| *PmTALE11*-R | TTTCTCGGCGGATTTCGTCT |
| *PmTALE13*-F | CTGTTGCGCAAGTACAGTGG |
| *PmTALE13*-R | GTGTCTGCTCCACCAATCCA |
| *PmTALE14*-F | TCCTGAAGTGGTGGCTAGGT |
| *PmTALE14*-R | CTCCATGAACTGGTCCAGCG |
| *PmTALE18*-F | CCGAATAGACTCCGAGTGCC |
| *PmTALE18*-R | TGGGTGCATGTGGTGGTTTA |
| *PmTALE19*-F | GCATATTGCTTGGCACTTGGG |
| *PmTALE19*-R | TAGCTCCTTGAGTTGCAGGTG |
| *Actin*-F | CCCTAAGGCTAACAGAGGAAACA |
| *Actin*-R | CATACATGGCAGGCACATTGAAG |

**Table S2.** Basic information of *P. mume* TALE gene family protein sequences

| **Protein name** | **Protein length （aa）** | **MW** | **pI** | **GRAVY** | **Instability coefficient** | **Subcellular localization** | **Homolog in Arabidopsis** |
| --- | --- | --- | --- | --- | --- | --- | --- |
| PmTALE1 | 814 | 89285.16 | 7.17 | -0.666 | 52.26 | Nucleus | BLH8 |
| PmTALE2 | 655 | 72053.19 | 7.21 | -0.68 | 43.44 | Nucleus | BLH1 |
| PmTALE3 | 648 | 72012.01 | 6.69 | -0.64 | 53.45 | Nucleus | RPL/BLH9 |
| PmTALE4 | 328 | 37181.68 | 5.11 | -0.663 | 40.61 | Nucleus | KNAT6 |
| PmTALE5 | 410 | 46374.53 | 6.33 | -0.856 | 54.84 | Nucleus | KNAT3 |
| PmTALE6 | 386 | 44011.72 | 6.13 | -1.011 | 49.83 | Nucleus | KNAT1 |
| PmTALE7 | 568 | 63492.85 | 6.43 | -0.502 | 52.93 | Nucleus | ATH1 |
| PmTALE8 | 162 | 17988.86 | 4.29 | -0.583 | 47.44 | Nucleus | KNATM |
| PmTALE9 | 605 | 67788.56 | 5.66 | -0.669 | 51.34 | Nucleus | BLH3 |
| PmTALE10 | 465 | 51866.25 | 5.79 | -0.452 | 53.61 | Nucleus | BLH11 |
| PmTALE11 | 353 | 40407 | 5.09 | -0.727 | 52.48 | Nucleus | KNAT6 |
| PmTALE12 | 143 | 16760.82 | 6.31 | -0.916 | 40.68 | Nucleus | KNAT6 |
| PmTALE13 | 383 | 42993.1 | 6.3 | -0.737 | 48.03 | Nucleus | STM |
| PmTALE14 | 329 | 36961.85 | 6.53 | -0.629 | 43.28 | Nucleus | STM |
| PmTALE15 | 656 | 72356.61 | 9.52 | -0.295 | 41.64 | Nucleus | RPL/BLH9 |
| PmTALE16 | 679 | 74747.92 | 5.83 | -0.674 | 45.62 | Nucleus | BLH7 |
| PmTALE17 | 283 | 32225.21 | 6.46 | -0.747 | 60.58 | Nucleus | KNAT7 |
| PmTALE18 | 704 | 79215.6 | 6.15 | -0.832 | 52 | Nucleus | BEL1 |
| PmTALE19 | 108 | 12042.49 | 4.63 | -0.602 | 56.46 | Nucleus | KNATM |
| PmTALE20 | 710 | 77740.23 | 6.83 | -0.683 | 44.83 | Nucleus | BLH1 |
| PmTALE21 | 616 | 66677.09 | 6.99 | -0.603 | 56.54 | Nucleus | BLH2 |
| PmTALE22 | 358 | 40833.49 | 5.61 | -0.795 | 54.2 | Nucleus | KNAT3 |
| PmTALE23 | 354 | 39734.49 | 5.45 | -0.667 | 41.21 | Nucleus | KNAT6 |

**Table S3.** Protein name and gene ID comparison table of *P. armeniaca* and *P. persica* TALE gene family members

| **Protein name** | **Protein ID** | **Protein name** | **Protein ID** |
| --- | --- | --- | --- |
| PaTALE1 | CAB4264336.1 | PpTALE1 | XP_007202255.1 |
| PaTALE2 | CAB4265252.1 | PpTALE2 | XP_007204251.1 |
| PaTALE3 | CAB4265659.1 | PpTALE3 | XP_007204284.1 |
| PaTALE4 | CAB4265660.1 | PpTALE4 | XP_007205519.2 |
| PaTALE5 | CAB4266104.1 | PpTALE5 | XP_007206432.1 |
| PaTALE6 | CAB4266139.1 | PpTALE6 | XP_007208166.1 |
| PaTALE7 | CAB4266824.1 | PpTALE7 | XP_007208167.1 |
| PaTALE8 | CAB4270851.1 | PpTALE8 | XP_007209425.1 |
| PaTALE9 | CAB4274148.1 | PpTALE9 | XP_007210325.1 |
| PaTALE10 | CAB4275719.1 | PpTALE10 | XP_007210588.2 |
| PaTALE11 | CAB4279795.1 | PpTALE11 | XP_007210953.2 |
| PaTALE12 | CAB4279960.1 | PpTALE12 | XP_007213815.2 |
| PaTALE13 | CAB4280764.1 | PpTALE13 | XP_007216163.2 |
| PaTALE14 | CAB4281428.1 | PpTALE14 | XP_007222130.1 |
| PaTALE15 | CAB4282749.1 | PpTALE15 | XP_007222132.1 |
| PaTALE16 | CAB4283057.1 | PpTALE16 | XP_007222468.2 |
| PaTALE17 | CAB4284612.1 | PpTALE17 | XP_007223034.1 |
| PaTALE18 | CAB4285146.1 | PpTALE18 | XP_007223047.1 |
| PaTALE19 | CAB4287031.1 | PpTALE19 | XP_007226414.2 |
| PaTALE20 | CAB4287573.1 | PpTALE20 | XP_007226843.2 |
| PaTALE21 | CAB4288767.1 | PpTALE21 | XP_020409919.1 |
| PaTALE22 | CAB4290105.1 | PpTALE22 | XP_020409927.1 |
|  |  | PpTALE23 | XP_020409930.1 |
|  |  | PpTALE24 | XP_020409933.1 |
|  |  | PpTALE25 | XP_020409979.1 |
|  |  | PpTALE26 | XP_020409980.1 |
|  |  | PpTALE27 | XP_020410200.1 |
|  |  | PpTALE28 | XP_020410201.1 |
|  |  | PpTALE29 | XP_020414171.1 |
|  |  | PpTALE30 | XP_020419768.1 |
|  |  | PpTALE31 | XP_020420269.1 |
|  |  | PpTALE32 | XP_020421595.1 |
|  |  | PpTALE33 | XP_020423343.1 |
|  |  | PpTALE34 | XP_020423576.1 |
|  |  | PpTALE35 | XP_020424621.1 |
|  |  | PpTALE36 | XP_020425206.1 |
